# Lectin-Based Study Reveals the Presence of Disease-Relevant Glycoepitopes in Bladder Cancer Cells and Ectosomes

**DOI:** 10.3390/ijms232214368

**Published:** 2022-11-19

**Authors:** Magdalena Surman, Magdalena Wilczak, Małgorzata Przybyło

**Affiliations:** Department of Glycoconjugate Biochemistry, Institute of Zoology and Biomedical Research, Faculty of Biology, Jagiellonian University in Krakow, 30-387 Kraków, Poland

**Keywords:** biomarkers, bladder cancer, ectosomes, glycosylation, lectins

## Abstract

Bladder cancer is a malignancy that remains a therapeutic challenge and requires the identification of new biomarkers and mechanisms of progression. Several studies showed that extracellular vesicles promote angiogenesis, migration and metastasis, and inhibit apoptosis in bladder cancer. This effect may depend on their glycosylation status. Thus, the aim of this study was to compare glycosylation profiles of T-24 urothelial bladder cancer cells, HCV-29 normal ureter epithelial cells, and ectosomes released by both cell lines using lectin blotting and flow cytometry. Ectosomes displayed distinct total and surface glycosylation profiles with abundance of β-1,6-branched glycans and sialilated structures. Then, it was investigated whether the glycosylation status of the T-24 and HCV-29 cells is responsible for the effect exerted by ectosomes on the proliferation and migration of recipient cells. Stronger proproliferative and promigratory activity of T-24-derived ectosomes was observed in comparison to ectosomes from HCV-29 cells. When ectosomes were isolated from DMJ-treated cells, the aforementioned effects were diminished, suggesting that glycans carried by ectosomes were involved in modulation of recipient cell function. HCV-29- and T-24-derived ectosomes also increased the viability and motility of endothelial HUVEC cells and Hs27 fibroblasts. This supports the hypothesis that ectosomes can modulate the function of various cells present in the tumor microenvironment.

## 1. Introduction

In recent years, a lot of attention has been paid to the role of extracellular vesicles (EVs) in intercellular communication. EVs represent a heterogeneous group of nanoscale structures surrounded by a phospholipid bilayer that are released in vivo and in vitro by almost all cell types. After their release to the intercellular space, EVs can be found circulating in various body fluids (e.g., blood, saliva, cerebrospinal fluid, and urine), or in conditioned cell culture media. Based on their size, biogenesis and molecular composition, EVs are classified into exosomes (30–150 nm of diameter), ectosomes (100–1000 nm) and apoptotic bodies (>1 μm). All three populations are known to modulate multiple biological processes in recipient cells, including gene expression, protein biosynthesis, the course of metabolic pathways, and intracellular signaling, by delivering biologically active molecules present in their cargo [1,2]. Regarding tumor-derived EVs, they can participate in pre-metastatic niche formation and favor tumor progression, metastasis, and immunosuppression [3]. The transfer of selected molecules (proteins, lipids, nucleic acids, etc.) to the recipient cell can occur through ligand–receptor interaction, EV fusion with the recipient cell membrane, as well as by endocytosis, phagocytosis, or micropinocytosis [4,5]. The effect exerted by EVs on the recipient cells directly depends on their molecular cargo that is determined by the type of cell they originate from. Moreover, EV molecular composition as well as the amount of EVs released undergo constant changes reflecting the most current state of the parental cell [6,7].

The precise mechanisms of sorting bioactive molecules into EVs and factors influencing the efficiency of the sorting process and EV incorporation into the recipient cells are still not well established. The endosomal sorting complex required for transport (ESCRT) is believed to play a central role in this process [8]. However, there is evidence that glycosylation can modulate not only the sorting [9,10,11,12,13] and incorporation of EVs [4,14,15,16,17,18,19,20], but also EV organ tropism [21].

Glycosylation is one of the most common modifications of membrane and secretory proteins as well as lipids. Numerous studies have shown that during neoplastic transformation, there are rapid changes in the biosynthesis of glycans followed by the appearance of so-called tumor associated carbohydrate antigens (TACAs) on the cell surface [22]. TACAs alter cell–cell and cell–extracellular matrix (ECM) interactions [23] and affect key biological processes, including cancer cell survival and proliferation, tumor invasion and metastasis, as well as immune evasion [24,25]. Changes in protein glycosylation are observed in various diseases not only at the cellular level, but also in isolated populations of EVs. It has been shown that exosomes released by cutaneous melanoma, leukemia, colon cancer [26], and ovarian cancer cells [27] display characteristic glycosylation patterns. Additionally, the presence of several cancer glycobiomarkers that are currently in clinical use has been confirmed in various EV populations [28].

Bladder cancer is the 10th most commonly diagnosed malignancy worldwide, with approximately 573,000 new cases and 213,000 deaths each year [29]. Due to the high rate of recurrence after tumor resection and the possibility of progression to more invasive and metastatic stages, bladder cancer still represents a therapeutic challenge. Therefore, there is a need to identify new biomarkers and mechanisms of bladder cancer progression for therapeutic purposes. So far, the role of EVs in bladder cancer has not been a subject of intensive research. Several groups have investigated the protein and nucleic acid content of bladder cancer-derived EVs, which led to the identification potential predictive biomarkers and proteins potentially involved in the metastatic process. Moreover, there have been studies demonstrating the direct involvement of bladder cancer-derived EVs in the promotion of angiogenesis, migration, and metastasis as well as in inhibition of apoptosis [30,31,32]. In our previous research, we showed that the glycosylation status of bladder cancer cells influences their adhesive and migratory properties [33,34]. Thus, the aim of this study was to compare the glycosylation profiles of T-24 urothelial bladder cancer cells, HCV-29 normal ureter epithelial cells, and ectosomes released by both cell lines. Then, we investigated whether the glycosylation status of the T-24 and HCV-29 cells is responsible for the effect exerted by ectosomes on the proliferation and migration of the recipient cells, i.e., HCV-29, T-24, fibroblasts, and human umbilical vein endothelial cells (HUVEC). These analyses were performed on HCV-29 and T-24 cells grown under standard conditions and in the presence of 1-deoxymannojirimycin (DMJ), which is a specific inhibitor of both ER and Golgi α1,2-mannosidases I, enzymes that are crucial for the maturation of N-glycans to the complex type [35].

## 2. Results

### 2.1. Characterization of Ectosome Samples

**Figure 1:**
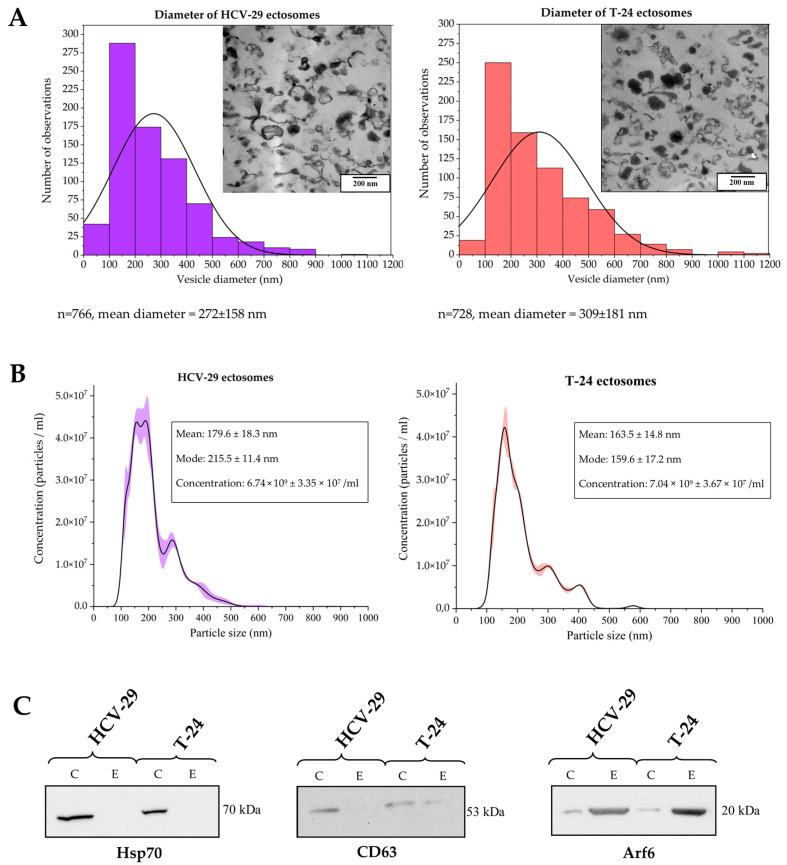
Characterization of ectosome samples isolated from conditioned media of T-24 urothelial bladder carcinoma and HCV-29 normal ureter epithelial cells. (**A**) Morphological characterization of HCV-29- and T-24-derived ectosomes by transmission electron microscopy (TEM). Size distributions are presented on histograms. Mean diameter ± standard deviation was calculated for all observed vesicles (n) from a given sample. (**B**) Nanoparticle tracking analysis (NTA) of HCV-29- and T-24-derived ectosomes. Results from five independent measurements for each cell line are presented on graphs. The shaded area depicts standard deviation. (**C**) Representative Western blot of extracellular vesicle markers in whole-cell protein extracts (lines C) and ectosome samples (lines E). Fifty μg of proteins separated by 10% SDS-PAGE and transferred into PVDF membrane were probed with anti-Hsp70 (1:2000), anti-CD63 (1:2000) and anti-Arf6 (1:500) as primary antibodies and anti-mouse IgG-HRP (1:400) as a secondary antibody.

All EV-oriented studies require thorough sample characterization. In the present study, EVs were isolated from conditioned media from HCV-29 and T-24 cell cultures using low vacuum filtration (LVF) followed by 18,000× *g* centrifugation. Transmission electron microscopy (TEM) and nanoparticle tracking analysis (NTA) were used to evaluate sample purity/contamination and vesicle size. On TEM images, neither intact cells, cellular organelles, nor other cellular debris were observed (Figure 1A). Isolated populations of ectosomes were quite heterogeneous in size, and the most numerous EV subpopulation had a diameter in the range of 100–300 nm for both cell lines. Moreover, NTA analysis confirmed that the most abundant EV subpopulations were those with a diameter in the range of 100–300 nm (Figure 1B). Furthermore, Western blot was used to analyze the expression of chosen EV protein markers (Figure 1C). It revealed the absence or depletion of exosomal markers (i.e., CD63 and heat shock protein 70 (Hsp70)) and enrichment in ADP-ribosylation factor 6 (Arf6) (the protein involved in ectosome biogenesis and, for this reason, considered to be an ectosomal marker) in all obtained EV samples in comparison to the whole cell protein extracts. Due to the fact that most of the isolated EVs were in the predefined diameter range for ectosomes (100–1000 nm) and displayed a marker expression pattern that is specific for ectosomes, we considered that contamination of the isolated samples with exosomes was negligible, and the obtained samples were highly enriched in ectosomes.

### 2.2. Lectin Blotting Analysis of Total Glycosylation of HCV-29 and T-24 Cells, Their Membrane Protein Fractions, and Released Ectosomes

**Figure 2:**
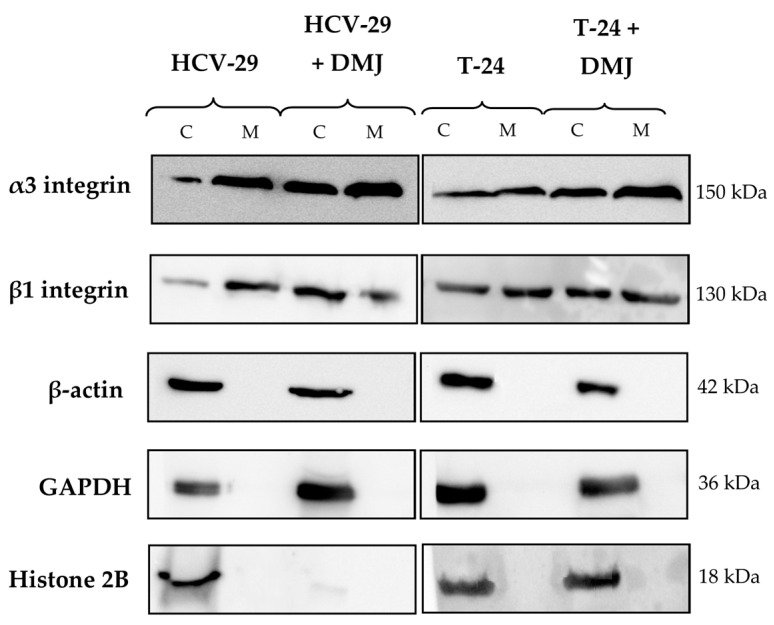
Assessment of the membrane fraction purity. On-blot immunodetection of proteins representative for each subcellular fraction was performed for the whole cell protein extracts (C) and the membrane fractions (M) obtained from HCV-29 and T-24 cell lines grown in control conditions and treated with DMJ. Loading controls: anti-α3 integrin subunit and anti-β1integrin subunit (for the membrane fraction), anti-β-actin (for the cytoskeletal fraction), anti-histone 2B (for the nuclear fraction), anti-GAPDH (for the cytosolic fraction).

In the present study, the whole cell protein extracts, the membrane fractions and ectosomal proteins from HCV-29 and T-24 cells were analyzed by lectin blotting to investigate their glycosignatures. Moreover, similar analysis was performed for cells treated with DMJ, a specific α-mannosidase I inhibitor that blocks the conversion of high mannose type to complex type N-oligosaccharides. First, the purity of the obtained membrane fractions was verified with the use of antibodies against proteins representative for the selected subcellular fractions (Figure 2). We confirmed the presence of two subunits of transmembrane integrin receptors, i.e., α3 and β1 subunits, in the whole cell extracts and in the corresponding membrane fractions. Actin, a major component of the cytoskeleton, was present only in the whole cell extracts and was detected as a band at molecular weight (MW) of 42 kDa. Core histone 2B, a nuclear protein, was detected only in the whole cell extracts at MW of 14 kDa. GAPDH was chosen as a loading control for the cytosolic fraction and detected in the whole cell extracts at MW of 37 kDa. Taken together, the isolated membrane fractions did not contain proteins specific for other subcellular fractions, and therefore they were used for further analysis.

**Figure 3:**
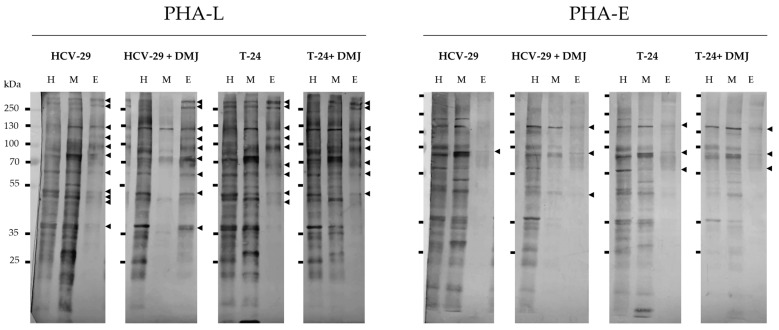
Detection of glycoproteins possessing β1,6-branched tri- and/or tetraantennary complex type N-glycans (PHA-L-positive) and bisecting GlcNAc bound to the core mannose of complex type N-glycans (PHA-E-positive). Results of lectin blotting with PHA-L and PHA-E are shown in the figure. Controls with the competitive sugars are shown in Supplementary Data (Figure S1). The major specific bands were indicated on the right with arrowheads. H, the whole cell protein extract; M, the membrane fraction; E, the ectosomal fraction.

**Figure 4:**
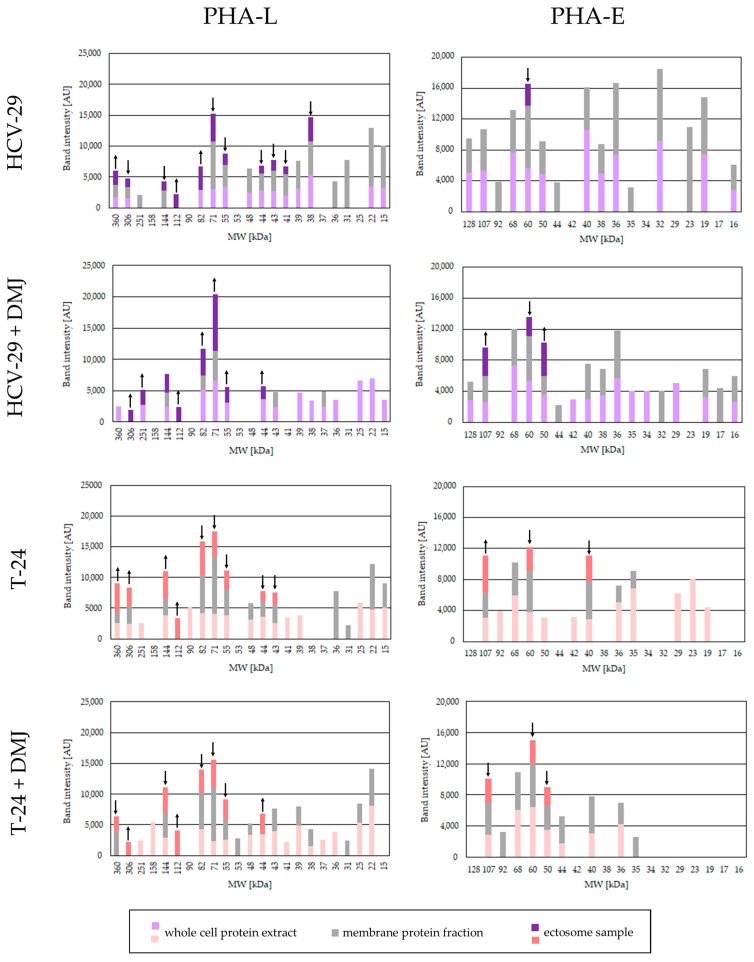
Densitometric analysis of PHA-L- and PHA-E-positive bands. The intensity of detected bands was expressed in arbitrary units (AU) as a peak area on respective densitogram. Each bar represents the intensity of corresponding bands in the whole cell protein extracts, membrane fractions and ectosome samples from each cell line. ↑ and ↓ denote a significant enrichment or depletion, respectively, of a given glycoepitope in the ectosomal fraction compared to membrane fraction. The values from control lectin blots with blocking solutions were subtracted beforehand.

On-blot reaction with PHA-L lectin confirmed the presence of β1,6-branched tri- and/or tetraantennary complex type N-glycans in the whole cell extracts and the membrane fractions from both HCV-29 and T-24 cells (Figure 3 and Figure 4). The number of PHA-L-positive bands was slightly higher for T-24 lysate than for HCV-29 lysate (16 vs. 13). In addition, the intensities of most bands detected for T-24 lysate (except for the band at 43 kDa) were higher than intensities of the corresponding bands from HCV-29 lysate. For the membrane fractions, this tendency was reversed, and a higher number of PHA-L positive bands was visualized for the membrane fraction from HCV-29 cells. Regarding the band intensities, no determined pattern was observed. Nearly half of the corresponding bands were randomly enriched in HCV-29 membrane fraction, whereas the other half was enriched in T-24 membrane fraction. Finally, 11 ectosomal protein bands were detected for HCV-29-derived ectosomes, and nine for T-24-derived ectosomes, suggesting that glycoproteins with β1,6-branched tri- and/or tetraantennary complex type N-glycan are abundantly present in ectosomes regardless of their origin. However, only two bands for HCV-29-derived ectosomes and four for T-24-derived ectosomes were enriched in comparison to the corresponding bands from the membrane fractions.

After DMJ-treatment, the number of PHL-positive bands in HCV-29 whole cell extract slightly increased (13 vs. 15), with only three of them showing decreased intensity (Figure 3 and Figure 4). The DMJ effect was, however, more prominent for HCV membrane fractions, where the number of bands decreased from 16 to 5, and all of them also showed decreased intensity when compared to the untreated control (Figure 3 and Figure 4). For the whole cell extract and membrane fraction from T-24 cells, the number of observed bands despite DMJ-treatment remained the same, but some bands with a lower intensity than in the untreated control were detected (Figure 3 and Figure 4). This lack of a uniform decrease in the intensity of PHA-L reaction post DMJ-treatment might be due to the ongoing process of protein turnover within the cells, the rates of which are different for individual glycoproteins. Regarding ectosomes obtained post DMJ treatment, a slight reduction in the number of PHA-L-positive bands was observed (Figure 3 and Figure 4). However, bands detected for ectosomes from DMJ-treated HCV-29 cells were enriched in comparison to corresponding bands from parental membrane fractions, and they also showed higher intensity than corresponding bands from the untreated control. This suggests that glycans recognized by PHA-L lectin were preferentially recruited to these ectosome, despite DMJ treatment.

PHA-E lectin was used to detect proteins possessing bisecting GlcNAc in their complex type N-glycans. In the whole cell extract and membrane fraction from HCV-29 cells protein bands were detected at 128, 107, 68, 60, 50, 40, 38, 36, 32, 19, and 16 kDa, mostly with similar or slightly decreased intensity in the membrane fraction (Figure 3 and Figure 4). Additional bands appeared in the HCV-29 membrane fraction at 92, 44, 35, and 23 kDa but not in the whole cell extracts, most likely due to their low abundance. Only one HCV-29-derived ectosomal band was detected at 60 kDa with lower intensity than the corresponding band in the membrane fraction (Figure 3 and Figure 4). In the case of T-24 cells, fewer PHA-E-positive bands were observed for both, the whole cell extract and membrane fraction, in comparison to HCV-29 cells (Figure 3 and Figure 4). Moreover, analogous bands that were visualized on blot for both cell lines showed a lower intensity in case of tumorous T-24 cells. That might be in agreement with the fact that bisecting GlcNAc is known to suppress tumor metastasis. Finally, three bands were visualized for T-24-derived ectosomes at 107, 60, and 40 kDa (Figure 3 and Figure 4). Only the band at 107 kDa displayed higher intensity than the corresponding band in the membrane fraction, suggesting that bisecting GlcNAc was not crucially involved in protein sorting into ectosomes.

As expected, after DMJ-treatment, a slightly weaker reaction with PHA-E lectin was observed for the whole cell extracts and membrane fractions from HCV-29 and T-24 cells (Figure 3 and Figure 4). For HCV-29 cells, the intensity of majority of protein bands was decreased in comparison to corresponding bands from the untreated control. However, few additional bands appeared at 42, 34, 29, and 17 kDa. In the case of DMJ-treated T-24 cells, fewer PHA-E-positive bands were observed for both the whole cell extract and membrane fraction in comparison to HCV-29 cells, and most of them displayed lower intensity than corresponding bands from the untreated control. Regarding ectosomes from both DMJ-treated HCV-29 and T-24 cells, three protein bands were observed on blots (Figure 3 and Figure 4). However, only the band at 50 kDa in HCV-29-derived ectosomes showed higher intensity than the band in the corresponding membrane fraction.

**Figure 5:**
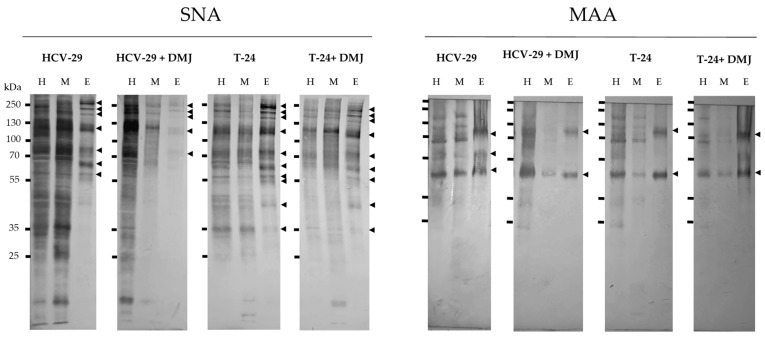
Detection of glycoproteins carrying α2,6-linked (SNA-positive) and α2,3-linked (MAA-positive) sialic acids. Results of lectin blotting with SNA and MAA are shown in the Figure. Controls with the competitive sugars are shown in Supplementary Data (Figure S2). The major specific bands were indicated on the right with arrowheads. H, the whole cell protein extract; M, the membrane fraction; E, the ectosomal fraction.

**Figure 6:**
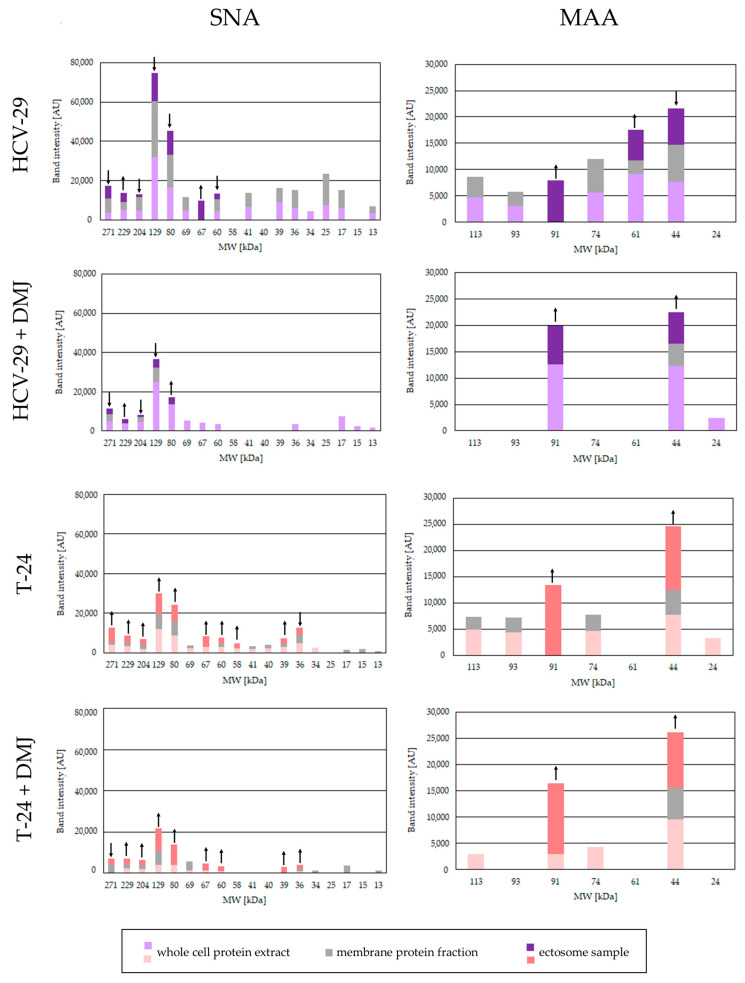
Densitometric analysis of SNA- and MAA-positive bands. The intensity of detected bands was expressed in arbitrary units (AU) as a peak area on respective densitogram. Each bar represents the intensity of corresponding bands in the whole cell protein extracts, membrane fractions and ectosome samples from each cell line. ↑ and ↓ denote a significant enrichment or depletion, respectively, of a given glycoepitope in the ectosomal fraction compared to membrane fraction. The values from control lectin blots with blocking solutions were subtracted beforehand.

Regarding sialylation, MAA and SNA lectins were used to detect α2,3- and α2,6-linked sialic acid, respectively. Several MAA-positive bands in HCV-29 whole cell extracts at 113, 93 74, 61 and 44 kDa were revealed (Figure 5 and Figure 6). Five corresponding bands were also visualized for HCV-29 membrane fractions, though they showed a lower intensity (Figure 5 and Figure 6). For T-24 whole cell lysates and membrane fractions, a similar band pattern was observed with the exception of 61 kDa band, whereas 24 kDa appeared (Figure 5 and Figure 6). Regarding ectosomes, for the HCV-29-derived sample, 61 and 44 kDa bands appeared with higher intensity than in the corresponding membrane fraction (Figure 5 and Figure 6). Moreover, a 91 kDa band appeared that was observed neither in lysate nor in the membrane fraction. For T-24 ectosomes, a band at 44 kDa was detected that showed enrichment in comparison to the corresponding band in the membrane fraction (Figure 5 and Figure 6). Moreover, as for the HCV-29-derived sample, an additional band at 91 kDa appeared.

After DMJ treatment, the number of MAA-positive bands visualized for HCV-29 whole cell extract and membrane fraction was reduced in comparison to the untreated control (Figure 5 and Figure 6). However, the intensity of detected bands (91, 44, and 24 kDa) was higher despite the treatment. On the other hand, both the number of bands and their intensity was reduced for the whole cell extract, and in particular for the membrane fractions of T-24 cells (Figure 5 and Figure 6). For ectosome samples derived post treatment from HCV-29 and T-24 cells, two bands were detected at 91 and 44 kDa. In both cases, band intensity was higher than for the corresponding bands in ectosomes from untreated cells.

The second lectin, SNA was used for detection of α2,6-linked sialic acid. The intensities of entire on-blot reactions for HCV29- and T-24-derived samples were considerably higher than in the analogous reaction with MAA lectin (Figure 5 and Figure 6). This suggests that glycans containing α2,6-linked sialic acid are more abundantly expressed by cells used for experiments than α2,3-linked sialic acid. Similar numbers of bands were detected in the whole cell extracts and membrane fractions from both cell lines, and the most prominent ones were at 129 and 80 kDa (Figure 5 and Figure 6). Regarding ectosomes, more bands (10 vs. 7) were detected for T-24 ectosomes (Figure 5 and Figure 6). Moreover, in the case of T-24-derived ectosomes, most of the detected bands showed higher intensity than the corresponding band in the membrane fraction. This might suggest that glycans bearing α2,6-linked sialic acid are to some degree preferentially sorted to ectosomes by cancerous bladder cells, but not by non-transformed cells.

After DMJ treatment, reduced numbers of SNA-positive bands and their intensities were observed on blot for both HCV-29- and T-24-derived samples (Figure 5 and Figure 6). The most substantial reduction was observed for membrane fractions from both cell lines. Regarding ectosomes, bands detected for T-24 ectosomes (from treated cells) remained enriched compared to the parental cell membrane fractions. On the other hand, HCV-29 ectosomal bands did not show a higher intensity than those from the corresponding membrane fraction.

**Figure 7:**
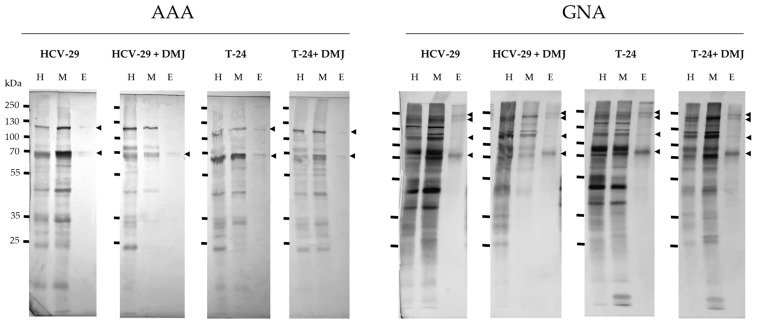
Detection of glycoproteins having fucose (AAA-positive) and mannose (GNA-positive) residues. Results of lectin blotting with AAA and GNA are shown in the Figure. Controls with the competitive sugars are shown in Supplementary Data (Figure S3). The major specific bands were indicated on the right with arrowheads. H, the whole cell protein extract; M, the membrane fraction; E, the ectosomal fraction.

**Figure 8:**
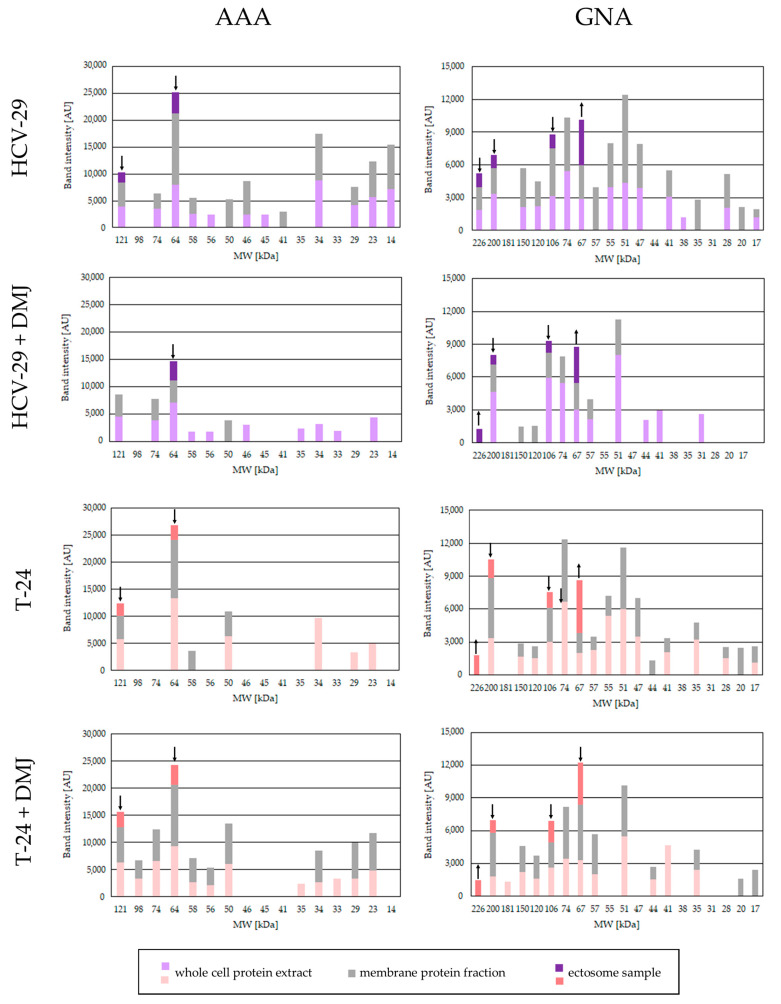
Densitometric analysis of AAA- and GNA-positive bands. The intensity of detected bands was expressed in arbitrary units (AU) as a peak area on respective densitogram. Each bar represents the intensity of corresponding bands in the whole cell protein extracts, membrane fractions and ectosome samples from each cell line. ↑ and ↓ denote a significant enrichment or depletion, respectively, of a given glycoepitope in the ectosomal fraction compared to membrane fraction. The values from control lectin blots with blocking solutions were subtracted beforehand.

The presence of proteins possessing fucose residues was revealed by the reaction with AAA lectin. AAA binds fucose with α1,6-linkage to the proximal GlcNAc residue as well as Galβ1,4(Fucα1,3) GlcNAc sequence. For HCV-29 whole cell extract and membrane fraction 9 pairs of the corresponding bands were detected, mostly with a higher intensity in the membrane fraction (Figure 7 and Figure 8). Additionally, two bands appeared only in HCV-29 lysate i.e., 56 and 45 kDa, and two were detected only in the membrane fraction i.e., 50 and 41 kDa. Far less bands were detected for T-24 whole cell extract (only 6) and membrane fraction (4 bands, respectively) (Figure 7 and Figure 8). For both ectosomal samples, two bands at 121 and 64 kDa were detected with a higher intensity in HCV-29-derived ectosomes (Figure 7 and Figure 8). However, intensities of all ectosomal bands were lower than those of the corresponding bands from the parental membrane fractions. This suggests that fucose residues within glycan structures are not crucial for protein sorting into ectosomes.

DMJ-treatment reduced the number of AAA-positive bands and their intensities observed on blot for HCV-29 samples (Figure 7 and Figure 8). The reduction was the most substantial for membrane fraction. Surprisingly, T-24 samples from DMJ-treated cells showed the opposite tendency (Figure 7 and Figure 8). Observed differences in response to DMJ between normal and cancerous cells might be due to the availability of GDP-fucose donor, abnormal expression of fucosylotransferases/fucosidases, and/or the availability of their substrates. Finally, in ectosomal samples isolated from cells post DMJ-treatment, the same bands with similar intensity were observed as in the untreated control, except for the 121 kDa band in HCV-29 ectosomes that was not detected.

The last reaction with GNA lectin–binding high-mannose and hybrid type glycans revealed similar binding pattern for all HCV-29 and T-24 samples (Figure 7 and Figure 8). Multiple GNA-positive bands were detected in HCV-29 and T-24 whole cell extracts and membrane fractions. In most cases, the intensity of the corresponding bands was higher for HCV-29 samples, suggesting that cancerous cells were less enriched in mannose-bearing glycans. Regarding ectosomes, four bands were detected for both HCV-29- and T-24-derived samples at 226, 200, 106, and 67 kDa, all with similar intensities. Nevertheless, only the bands at 67 kDa showed the enrichment in comparison to the corresponding bands visualized for the parental membrane fractions.

DMJ-treatment was expected to increase the content of high-mannose and hybrid type glycans. In whole cell extract and membrane fraction from DMJ-treated HCV-29 cells number of bands decreased (Figure 7 and Figure 8). However, the intensity of the remaining bands (mostly for lysate) increased in comparison to the untreated control. For the whole cell extract and membrane fraction from DMJ-treated T-24 cells, the reduction in the number of GNA-positive bands was lower. Some had the decreased intensity in comparison to untreated control, but the increased intensity was observed for bands at 150, 120, and 67 kDa (lysate and membrane fraction), 44 and 41 kDa (lysate only), and 57, 35, and 17 kDa (for membrane fraction only). The observed differences in DMJ-response seem to be cell line specific and dependent on the rate of protein turnover for particular proteins. As for the ectosomes from DMJ-treated cells, the same four GNA-positive bands were detected that appeared earlier in control samples. Almost all of them (except 106 kDa for T-24 ectosomes) showed similar or only slightly lower intensity than in ectosomes from the untreated cells. This suggests that mannose residues are also neither crucial nor required for protein sorting into ectosomes.

### 2.3. Surface Glycosylation of HCV-29 and T-24 Cells and Released Ectosomes

The same panel of lectins (i.e., AAA, GNA, SNA, MAA, PHA-E, and PHA-L) was later used for the flow cytometry staining of HCV-24 and T-24 cells as well as ectosomes derived from them. That allowed us to examine the extent to which the revealed enrichment or depletion of given glycoepitopes in isolated ectosomes in comparison to the parental cell membranes is related to the surface (plasma membrane-associated).

**Figure 9:**
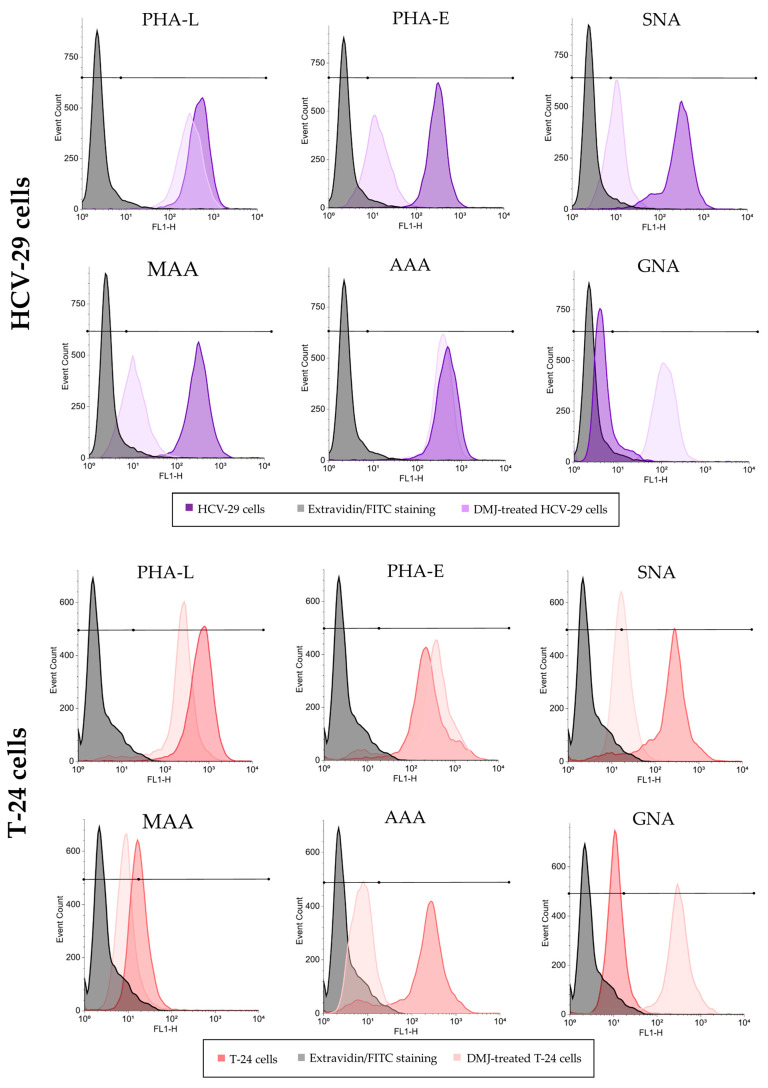
Flow cytometry analysis of surface glycosylation of HCV-29 and T-24 cells grown in control conditions and treated with DMJ. Histograms for MAA-, SNA-, GNA-, AAA-, PHA-E- and PHA-L-positive cells. Colored areas represent stainings with a panel of six lectins, while grey histograms represent background fluorescence of unspecific binding of FITC-extravidin. The X axis shows log fluorescence intensity, Y axis shows the number of events.

**Figure 10:**
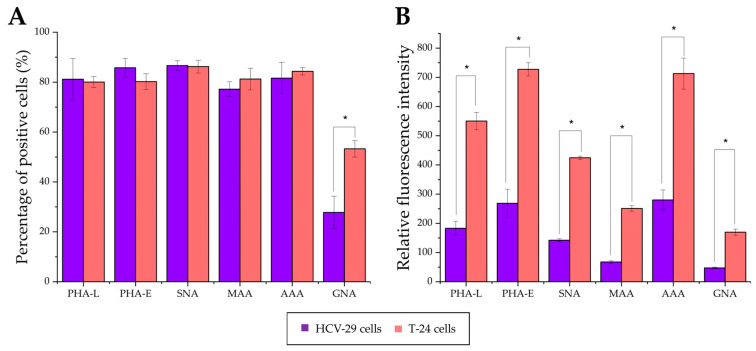
Flow cytometry analysis of surface glycosylation of HCV-29 and T-24 cells grown in control conditions. (**A**). Diagram showing the percentage of positive cells in each staining. (**B**). Diagram showing the relative fluorescence intensity (RFI) for each staining. *—denotes statistically significant differences.

Approximately between 77% and 86% of HCV-29 and T-24 cells possessed α2,3- and α2,6- linked sialic acids, fucose residues, bisecting GlcNAc in complex type N-glycans, as well as β1,6-branched tri- and/or tetraantennary complex type N-glycans as revealed by positive staining with MAA, SNA, AAA, PHA-E, and PHA-L, respectively (Figure 9 and Figure 10). No statistical differences were shown for the aforementioned staining when comparing HCV-29 and T-24 cells. Only the reaction with GNA revealed a higher percentage of cells bearing mannose residues in case of tumorous T-24 cells (53% vs. 28% for HCV-29%) and, in general, the number of GNA-positive cells was much smaller than in other lectin staining. Although the substantial differences in the percentage of positive cells were observed only for GNA staining, the measurement of relative fluorescence intensity (RFI) revealed a greater diversity related to the relative amount of respective glycoepitopes on the cell surface. All six evaluated epitopes were more abundantly present on the cell surface of tumorous T-24 cells compared to normal HCV-29 cells.

**Figure 11:**
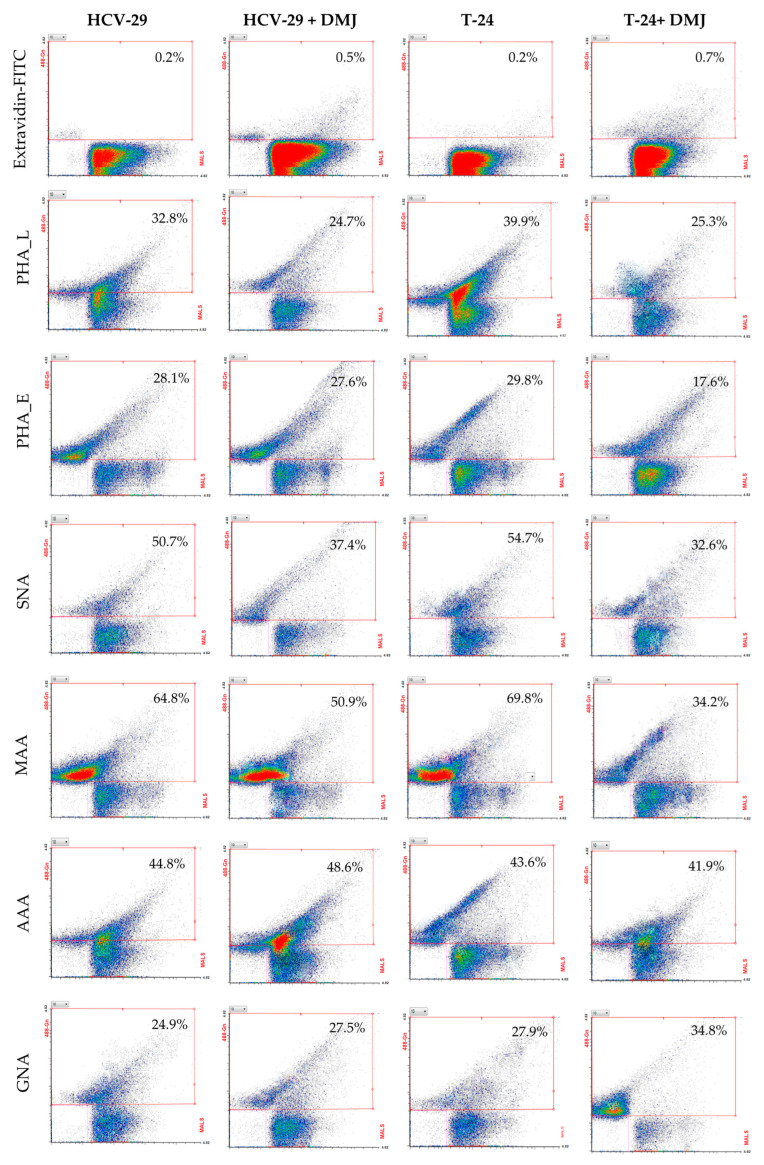
Flow cytometry analysis of surface glycosylation of ectosomes derived from HCV-29 and T-24 cells grown in control conditions or treated with DMJ. Dot plots for MAA-, SNA-, GNA-, AAA-, PHA-E- and PHA-L-positive ectosomes. Results for control secondary extravidin-FITC conjugate staining are also provided. The X axis shows values obtained at medium angle light scatter (MALS), Y axis shows log fluorescence intensity measured on 488Gn laser.

**Figure 12:**
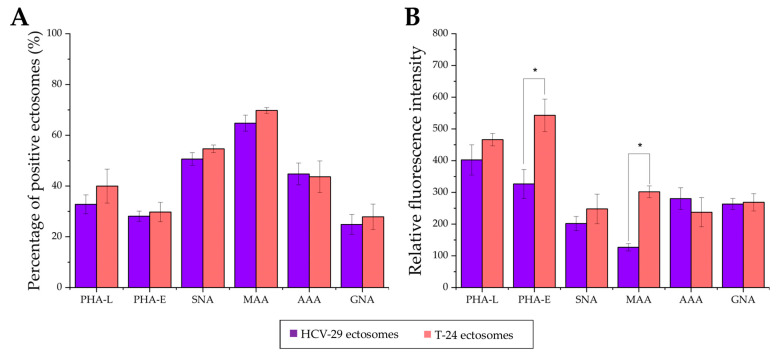
Flow cytometry analysis of surface glycosylation of ectosomes derived from HCV-29 and T-24 cells grown in control conditions or treated with DMJ. (**A**). Diagram showing the percentage of positive ectosomes in each staining calculated by the subtraction of the value for the unspecific binding (secondary FITC-extravidin staining) from the raw value for each lectin staining. (**B**). Diagram showing the relative fluorescence intensity (RFI) for each staining. *—denotes statistically significant differences.

Considering HCV-29- and T-24-derived ectosomes, the percentage of lectin-positive vesicles was decreased in comparison to the analogous stainings of parental cells (Figure 11 and Figure 12). Moreover, no differences in percentage were observed for the aforementioned staining when comparing HCV-29- and T-24-derived ectosomes. The highest percentage of positive vesicles was observed for MAA staining (~65% and 69% for HCV-29- and T-24-derived ectosomes, respectively), and the lowest for GNA staining (~25% and 28%). Regarding RFI, ectosomes showed a similar tendency relative to the cells of their origin i.e., higher RFI values in particular staining for ectosomes derived from tumorous T-24 cells compared to HCV-29 normal cells (Figure 11 and Figure 12). Nevertheless, statistically significant differences were noted only for MAA and PHA-E staining.

**Figure 13:**
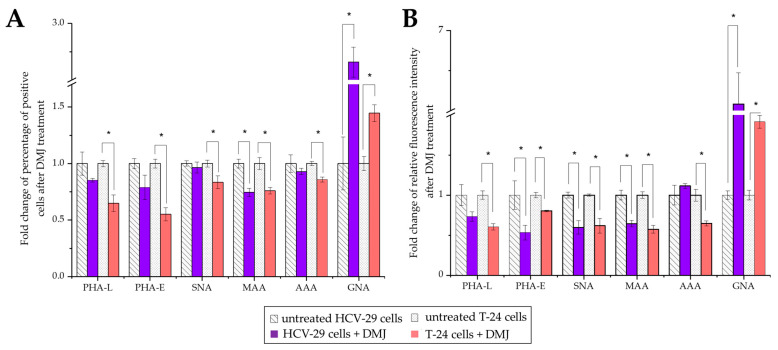
Flow cytometry analysis of surface glycosylation of HCV-29 and T-24 cells grown in control conditions or treated with DMJ. (**A**). Diagram showing the fold change of percentage of positive cells in each staining. (**B**). Diagram showing the fold change of relative fluorescence intensity (RFI) for each staining. *—denotes statistically significant differences.

Furthermore, DMJ treatment of HCV-29 and T-24 cells induced significant changes in cell surface glycan composition (Figure 9 and Figure 13). As expected, GNA staining revealed ~2.6- and ~1.5-fold increases in the percentage of positive HCV-29 and T-24 cells, respectively. That corresponds with the increase in the amount of high mannose glycans on the cell surface revealed also by RFI value change (~6-fold increase for HCV cells, ~1.9-fold increase for T-24 cells). Such results might suggest that HCV-29 were more sensitive to DMJ treatment. However, the remaining five stainings showed the loss of particular epitopes mostly by T-24 cells. The most significant decrease in the percentage of positive T-24 cells was observed for PHA-E and PHA-L staining, whereas the highest decrease in RFI was revealed for MAA and SNA staining. On the contrary, HCV-29 cells decreased in both percentages of positive cells and RFI was observed only for MAA staining, whereas RFI decreased also in PHA-E and SNA stainings.

**Figure 14:**
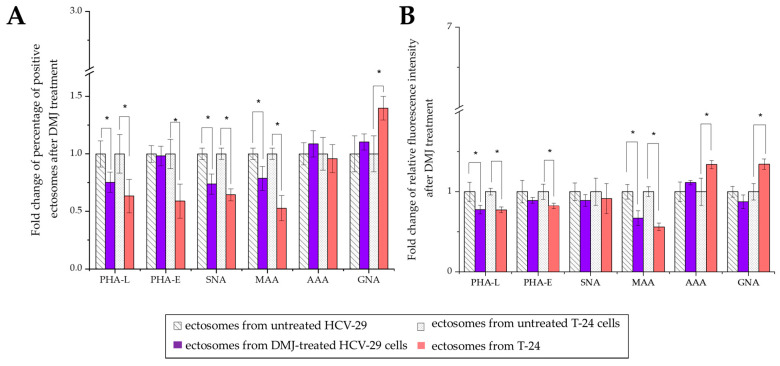
Flow cytometry analysis of surface glycosylation of ectosomes derived from HCV-29 and T-24 cells grown in control conditions or treated with DMJ. (**A**). Diagram showing the fold change of percentage of positive ectosomes in each staining. (**B**). Diagram showing the fold change of relative fluorescence intensity (RFI) for each staining. *—denotes statistically significant differences.

Regarding ectosomes isolated from DMJ-treated cells, the reaction with GNA revealed the increased percentage of positive vesicles and RFI for T-24-derived ectosomes but not HCV-29-derived ectosomes (Figure 11 and Figure 14). Similarly, only ectosomes derived from DMJ-treated T-24 cells showed changes in bisecting GlcNAc surface expression with decreased percentage of positive vesicles and RFI in PHA-E staining. On the other hand, ectosomes derived from both DMJ-treated cell lines displayed a decreased percentage of positive vesicles and RFI in MMA staining and PHA-L staining (Figure 11 and Figure 14). This suggests that changes in the content of α2,3- linked sialic acids and β1,6-branched tri- and/or tetraantennary complex type N-glycans are generally reflected on the surface of ectosomes released by DMJ-treated cell cells. Regarding α2,6- linked sialic acids, decreased percentages of SNA-positive vesicles were observed for both HCV-29- and T-24-derived ectosomes. However, these observations were not followed by decreased RFI values. Finally, the reaction with AAA did not reveal any changes in fucose content of ectosomes besides the increased RFI for T-24-ectosomes (Figure 11 and Figure 14). Since DMJ-treatment of T-24 cell decreased the fucose content in the parental cell, the observed increase in ectosomes seems untypical and might be cell-line specific. Overall, fucose residues on cell surface glycoproteins are unlikely to be determinants of where ectosomes are formed within the outer cell membrane and are unlikely to be involved in protein sorting into ectosomes.

### 2.4. Functional Effect of HCV-29- and T-24-Derived Ectosomes on Recipient Cells Viability and Migratory Properties

**Figure 15:**
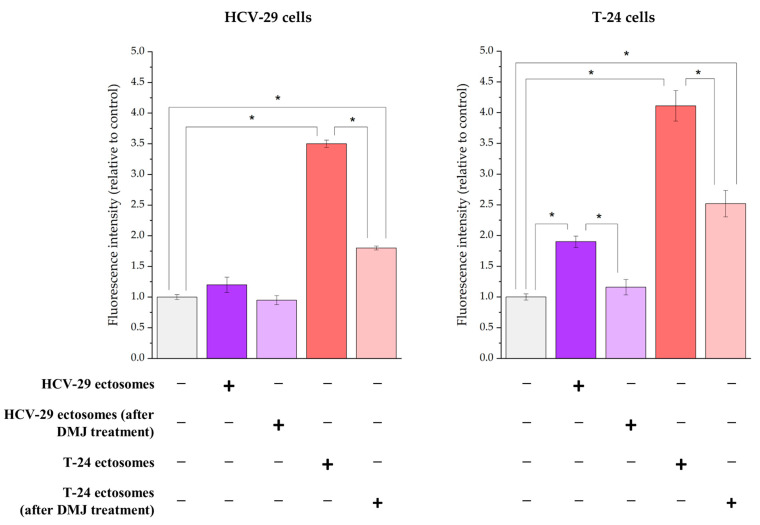
Effect of incubation of HCV-29 normal ureter epithelial cells (**left panel**) and T-24 urothelial bladder carcinoma cells (**right panel**) with ectosomes released by these cell lines without or after DMJ treatment on cell viability. Alamar Blue cell viability assay was carried out after 18 h of incubation with ectosomes. All experiments were conducted in triplicate. “*” denotes statistically significant differences (Tukey’s post-hoc test, *p*-value < 0.05).

Furthermore, the functional effect of HCV-29- and T-24-derived ectosomes as well as aberrantly glycosylated ectosomes (derived from DMJ-treated cells) on recipient cells was assessed using an Alamar Blue cell viability assay (Figure 15). The addition of T-24-derived ectosomes (60 µg of protein) caused an over four-fold increase in the measured fluorescence intensity of recipient T-24 cells compared to the untreated controls, while its effect exerted on HCV-29 cells was significantly weaker (3.5-fold). On the other hand, although HCV-29-derived ectosomes (60 µg of protein) increased the viability of recipient T-24 cells (by approximately two-fold), they did not induce any changes in the viability of HCV-29 cells. This suggests stronger pro-proliferative action of tumor-derived T-24 ectosomes compared to ectosomes from normal HCV-29 cells. Moreover, HCV-29 cells were less responsive to treatment with ectosomes. When added ectosomes were isolated from DMJ-treated cells, the aforementioned effects were diminished by approximately 50%, although the cell viability was still increased in comparison to control conditions (Figure 15). The only exception were HCV-29 cells treated with HCV-29-derived ectosomes (post DMJ treatment), where the levels of fluorescence did not show any differences compared to control. This suggests that glycans carried by ectosomes are involved in the modulation of recipient cell viability, and their alterations induced by DMJ diminished the pro-proliferative effect of ectosome treatment.

**Figure 16:**
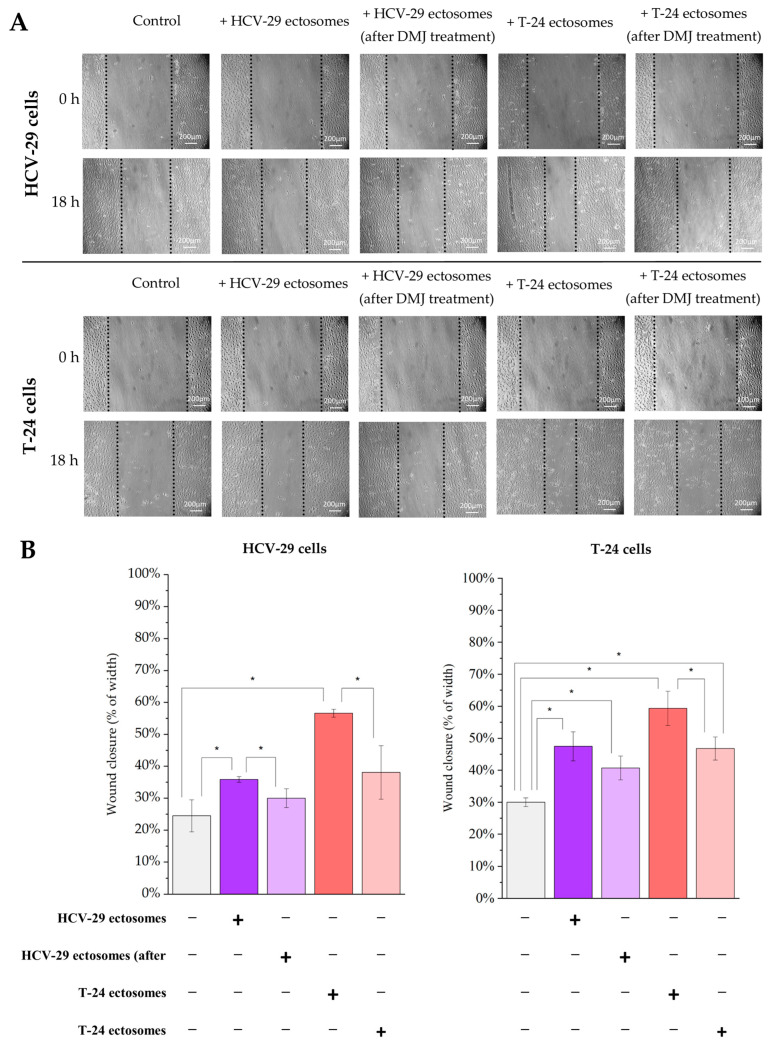
Effect of incubation of HCV-29 normal ureter epithelial cells (**left panel**) and T-24 urothelial bladder carcinoma (**right panel**) cells with ectosomes released by these cell lines without or after DMJ treatment on cell motility. Wound healing assay was performed after 18 h of incubation with ectosomes. (**A**). Representative images were taken at 0 h and at 18 h. (**B**). Graphs presenting the percentage rate of wound closure calculated from three repetitions. “*” denotes statistically significant differences (Tukey’s post-hoc test, *p*-value < 0.05).

Finally, migratory properties of HCV-29 and T-24 cells upon ectosome treatment were assessed in a wound healing assay (Figure 16). Approximately 24% of wound width closure was observed for normal HCV-29 cells in control conditions, while T-24 cells displayed a slightly higher rate of ~30%. When treated with T-24-derived ectosomes, both recipient cell lines showed an increase in wound width closure up to 60%, whereas the addition of HCV-29 ectosomes caused a slightly lower increase (from 24% to 35% for HCV-29 cells, and from 30% to 47% for T-24 cells). Regarding ectosomes from DMJ-treated cells, their altered glycosylation diminished the promigratory effect of ectosomes (Figure 16). The only exception was T-24 cells treated with HCV-29 ectosomes (post DMJ treatment), where the decrease in wound width closure was not statistically significant. Nevertheless, as in the viability assay, wound width closure rate remained increased in comparison to control conditions despite the use of ectosomes from DMJ-treated cells.

**Figure 17:**
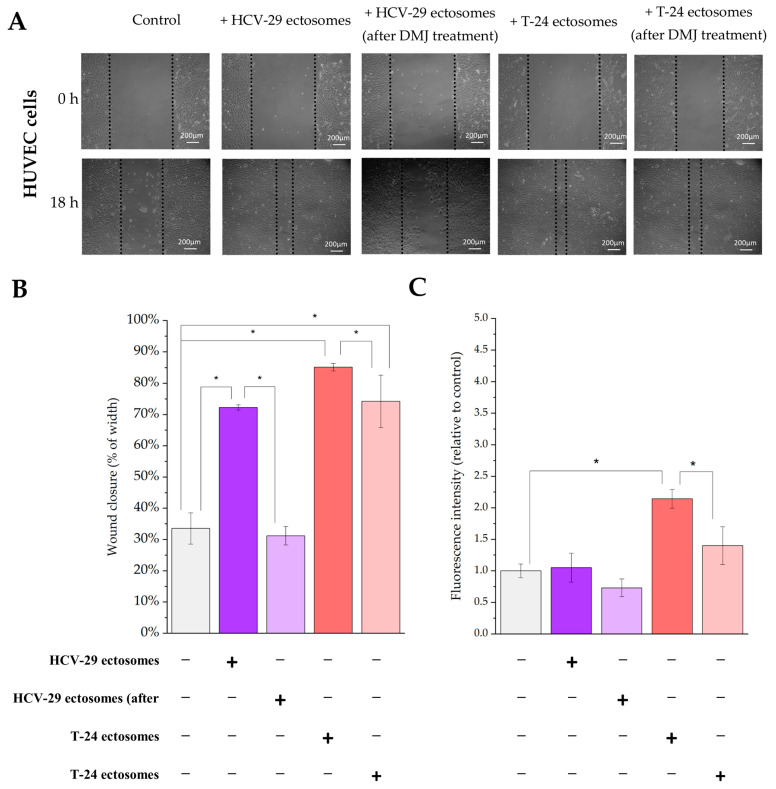
Effect of incubation of endothelial HUVEC cells of with ectosomes released by HCV-29 normal ureter epithelial cells and T-24 urothelial bladder carcinoma cells without or after DMJ treatment on cell viability and motility. Wound healing assay and Alamar blue assays was performed after 18 h of incubation with ectosomes. (**A**). Representative images from wound healing assay were taken at 0 h and at 18 h. (**B**). Graph presenting the percentage rate of wound closure calculated from three repetitions. (**C**). Graph presenting fluorescence intesity measeured in Alamar blue assay relative to control. “*” denotes statistically significant differences (Tukey’s post-hoc test, *p*-value < 0.05).

**Figure 18:**
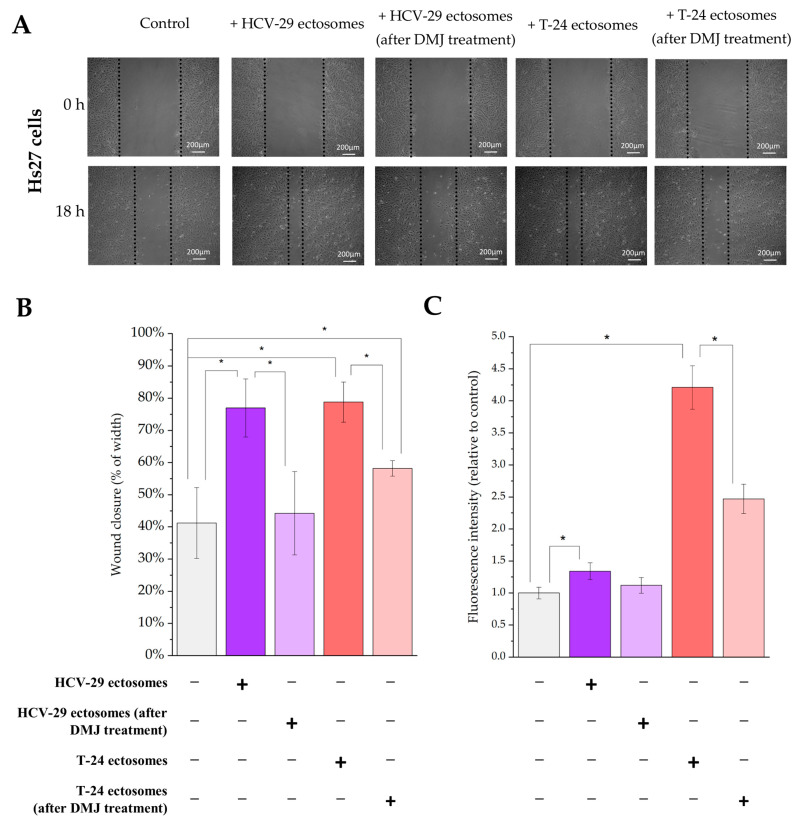
Effect of incubation of Hs27 fibroblasts of with ectosomes released by HCV-29 normal ureter epithelial cells and T-24 urothelial bladder carcinoma cells without or after DMJ treatment on cell viability and motility. Wound healing assay and Alamar blue assays was performed after 18 h of incubation with ectosomes. (**A**). Representative images from wound healing assay were taken at 0 h and at 18 h. (**B**). Graph presenting the percentage rate of wound closure calculated from three repetitions. (**C**). Graph presenting fluorescence intesity measeured in Alamar blue assay relative to control. “*” denotes statistically significant differences (Tukey’s post-hoc test, *p*-value < 0.05).

Similar functional tests were performed for endothelial HUVEC cells and Hs27 fibroblasts as recipient cells. For HUVECs, we observed an approximately two-fold increase in cell viability after addition of T-24-derived ectosomes, but not HCV-29-derived ectosomes (Figure 17C). In the wound healing assay, ectosomes from both cell lines significantly increased wound closure rate, to a greater degree in the case of T-24-derived ectosomes (from 32% for control condition to approximately 85% and 73% for T-24- and HCV-29-derived ectosomes, respectively) (Figure 17A,B). For Hs27 fibroblasts, we observed an over 4.2-fold increase in cell viability after addition of T-24-derived ectosomes, but only a 1.3-fold increase for HCV-29-derived ectosomes (Figure 17C). In the wound healing assay, ectosomes from both cell lines significantly increased wound closure rate to a similar degree from 40% for control condition to approximately 80% (Figure 18A,B). When ectosomes from DMJ-treated cells were added to HUVECs or Hs27 fibroblasts, the pro-proliferative impact was diminished only in case of ectosomes derived form DMJ-treated T-24 cells while promigratory properties decreased when ectosomes derived from both DMJ-treated cells were added (Figure 17 and Figure 18). In the case of Hs27 fibroblasts, the decrease in migratory properties was stronger.

Aforementioned results suggest that glycoproteins carried by ectosomes are one but not the only factor involved in ectosome–recipient cell interactions and subsequent biological processes which lead to increased cell motility. Moreover, the functional effect exerted by ectosomes may depend on the type of recipient cells since different responses were observed between recipient bladder cells, endothelial cells, and fibroblasts. Nevertheless, obtained results support the hypothesis that ectosome can modulate function of various cells present in the tumor microenvironment.

## 3. Discussion

### 3.1. Glycan Epitopes in Ectosomal Protein Sorting

Tumor-derived EVs are important mediators of intercellular communication, and their molecular content can alter the state and behavior of recipient cells at various levels. The composition of EV cargo very much resembles that of the parental cell. However, the specific sorting mechanisms for different molecular components occur during EV biogenesis. As a result of the sorting mechanism, subpopulations of EVs possess unique molecular profiles, among which several glycoepitopes can be distinguished. There is a well-documented protein sorting activity of ESCRT [8] that is involved in multivesicular body formation during biogenesis of exosomes. Similar constitutive mechanism has not been described for ectosomes and glycan-based protein sorting is one of the most extensively studied alternatives in that matter. Tumor-derived EVs have already been shown to be enriched in high mannose and complex type N-glycans, polylactosamine, and sialilated glycans (mainly having 2,6-linked sialic acids) [10,11,26,27]. However, the pattern of glycan depletion/enrichment of EV subpopulations seem to be cancer type-specific and require further investigation.

So far, the involvement of complex type N-glycan in EV protein sorting has been the most widely studied. In a study on OVMz cells, kifunensine (α-mannosidase I inhibitor, preventing the processing of high-mannose to complex type N-glycans) decreased the levels of several N-linked glycoproteins (CD63, LGALS3BP, L1CAM) in EVs, but did not affect the level of nonglycosylated annexin-I [11]. Another α-mannosidase I inhibitor–DMJ (also used in the present study) decreased the level of glycoprotein EWI-2 in exosomes, and the same effect was observed after N-glycosylation site-directed abrogation. In the same study, exosome enrichment in high-mannose glycans, poly-N-acetyllactosamine structures, and α2,6-linked sialic acids was also observed [10].

In the present study, we observed abundance of β1,6-branched tri- and/or tetraantennary complex type N-glycans (positive on-blot reaction with PHA-L) in ectosomes derived from both HCV-29 and T-24 cells. Bands detected for ectosomes derived from DMJ-treated HCV-29 cells were enriched in β1,6-branched tri- and/or tetraantennary complex type N-glycans in comparison to the parental membrane fractions, and they also showed a higher intensity than the corresponding bands from the untreated control. This suggests that glycans recognized by PHA-L lectin were preferentially recruited to these ectosomes despite DMJ treatment. On the other hand, DMJ treatment decreased percentage of PHA-L-positive ectosomes and RFI in flow cytometry analysis. Therefore, preferential sorting of β1,6-branched tri- and/or tetraantennary complex type N-glycans into ectosomes may not concern surface proteins. Regarding bisecting GlcNAc, we did not observe any significant on-blot enrichment of PHA-E-positive ectosomal bands in comparison to the parental membrane fractions. Moreover, in flow cytometry analysis, ectosomes derived from DMJ-treated T-24 cells showed decreased the surface expression of bisecting GlcNAc. This suggests that bisecting GlcNAc was not crucially involved in protein sorting into ectosomes. However, our previous studies on cutaneous and uveal melanoma-derived ectosomes revealed the enrichment in glycans with bisecting GlcNAc [36,37], pointing to a more cell- or cancer-specific character of glycan-based protein sorting.

Following up on sialilation of EV proteins, in a study by Jung et al. [38], β-galactoside α-2,6-sialiltransferase 1 (ST6Gal1) knock-out in the SW620 colorectal cancer cells increased tumor cell adhesion and migration. The observed effect was attributed to depletion of KAI1 tetraspanin in EVs, which is an inhibitor of crucial signaling pathways during cancer metastasis. Moreover, our previous study revealed selective enrichment of uveal melanoma-derived ectosomal proteins with α2,6-linked sialic acids, indicating that sialic acid residues may be significant for ectosome formation [37]. In the present study, α-2,6-linked sialic acids were detected in the reaction with SNA lectin. More SNA-positive bands were detected on-blot for T-24 ectosomes and most of them were enriched in comparison to the parental membrane fraction. Further, decreased percentages of SNA-positive vesicles were revealed for both HCV-29- and T-24-derived ectosomes by flow cytometry. However, these observations were not followed by decreased RFI values. This might suggest that glycans bearing α2,6-linked sialic acids are preferentially sorted to ectosomes by cancerous bladder cells but not by non-transformed cells.

Regarding α-2,3-linked sialic acids, fewer bands were detected on-blot for MAA staining than for SNA staining. However, for HCV-29- and T-24-derived ectosomes, detected bands showed the enrichment in comparison to the parental membrane fractions. Moreover, the intensities of the corresponding bands were higher after DMJ treatment than for ectosomes from the untreated cells. On the other hand, ectosomes from both DMJ-treated cell lines displayed a decreased percentage of MAA-positive vesicles and RFI in flow cytometry analysis. Similar to the observation from PHA-L staining, the involvement of α2,3-linked sialic acids in ectosomal protein sorting may vary for particular groups of proteins.

Furthermore, a recent study showed that the overexpression of α-(1,6)-fucosyltransferase (FUT8) by prostate cancer cells altered the proteome of EVs released by those cells [12]. A decreased number of ESCRTs and endocytosis-related components was observed, resulting in lower amounts of secreted EVs. Higher fucose content also led to the increased abundance of proteins associated with cell motility and cancer metastasis in the same EVs. Fucosylated glycans were also preferentially sorted into ectosomes released by cutaneous melanoma cells [36]. Results from the present study did not confirm the involvement of fucose residues in ectosomal protein sorting. Intensities of all AAA-positive bands from HCV-29- and T-24-derived ectosomes were lower than those of the corresponding bands from the parental membrane fractions and kept similar values after DMJ-treatment. Moreover, in flow cytometry analysis, no changes in fucose content of ectosomes were observed, besides the increased RFI for T-24-derived ectosomes.

Regarding mannose residues, HCV-29- and T-24-derived ectosomes showed no significant enrichment in on-blot GNA-staining in comparison to the parental membrane fractions. For ectosomes from DMJ-treated cells, similar or even slightly lower band intensity than in ectosomes from the untreated cells was observed. Further, the percentage of positive vesicles and RFI measured by flow cytometry was the lowest for GNA staining, while DMJ-treatment increased the percentage of positive vesicles and RFI only for T-24-derived ectosomes. This suggests that high mannose glycans are neither crucial nor required for protein sorting into ectosomes.

Finally, there is direct evidence that the sorting of particular proteins into EVs may not be glycan-dependent. The example is Lipocalin 2 (LCN2), a protein that is abundantly secreted in EVs and has one determined N-glycosylation site. The research on several cell lines showed that treatment with tunicamycin (N-glycosylation inhibitor) did not affect sorting of LCN2 into EVs, suggesting that there is another mechanism involved which is not related to glycan structures [39]. Moreover, our results from lectin stainings did not reveal a uniform enrichment/depletion pattern regarding both on-blot protein-bands and surface expression. Considering all the aforementioned studies, the role of glycan structures in EV protein sorting appears to be determined by a cell type. The selection of cell membrane domains where EVs are formed, and therefore the sorting of particular proteins into EVs, may be regulated by different glycan structures. Further, the glycan-based sorting mechanism may not be related to the entire protein EV cargo, but to particular groups of proteins.

### 3.2. Ectosomal Glycoproteins and Their Functional Role in Cancer

Aberrant protein glycosylation is one of the common features of cancer cells. Glycans, including neoglycoepitopes, can contribute to tumor cell dissociation and invasion, mainly by interfering with intercellular signaling and cell-cell adhesion. An increased number of β1,6-branched N-glycan structures generally impairs the adhesion of tumor cells and promotes their migratory phenotype. These β1,6-branched structures can be extended by terminal sialilation what alters cell–extracellular matrix interactions and further increase invasiveness of tumor cells. Moreover, the sialilated and fucosylated structures form the sialilated Lewis determinants that participate in the formation of metastases via interaction with selectins.

Regarding bladder cancer, previous studies on T-24 cells showed that swainsonine (α-mannosidase II inhibitor) treatment reduced the rate of T-24 cell migration on fibronectin and in wound healing assay. Moreover, the adhesion of swainsonine-treated HCV-29 and T-24 cells was increased on fibronectin and type IV collagen and laminin [33,34]. That indicates that β1-6 branched tri- and tetraantennary complex type N-glycans (particularly on cell adhesion molecules (CAMs) i.e., integrins and cadherins) can modulate adhesive and migratory properties of bladder cancer cells. Results from aforementioned research correspond with those obtained in the present study. We observed higher migratory rate for bladder cancer T-24 cells in comparison to normal HCV-29 cells in a wound healing assay. This can be indirectly attributed to a higher content of β1,6-branched structures in T-24 cells confirmed by both Western blot (band number) and flow cytometry with PHA-L lectin staining.

The function of cell CAMs in bladder cancer can also be regulated by their fucosylation status. Calreticulin has been shown to increase adhesion of bladder cancer cells by stabilizing mRNA for fucosyltransferase 1 (FUT-1), an enzyme responsible for α-1,2-linked fucosylation of β1 integrin [40]. Moreover, fucosyltransferase 7 (FUT-7) expression was positively correlated with the number of tumor-infiltrating lymphocytes, tumor cell proliferation, migration, and invasion rate, as well as occurrence of epithelial-mesenchymal transition (EMT) in bladder cancer cells [41]. EMT-related changes in N-glycosylation were also examined using HCV-29 cells [42]. Induction of EMT with transforming growth factor-beta (TGFβ) reduced the expression of α-mannosidase II. That was followed by decreased expression of complex type N-glycans, and increased expression of hybrid type N-glycans. Moreover, decreased expression of α-L-fucosidase in TGFβ-induced EMT was observed, which contributed to increased expression of fucosylated N-glycans. In the present study, the expression of fucosylated glycans was assessed with the use of AAA lectin. Total fucosylation analyzed on blot was weaker for T-24 cells. However, the surface expression of fucosylated glycans (RFI for AAA staining in flow cytometry analysis) was higher than for normal HCV-29. This suggests that increased fucosylation in bladder cancer cells may indeed concern mainly surface proteins, including CAMs, which may be responsible for the higher migratory rate of T-24 cells observed in the wound healing assay.

Furthermore, sialilation of various proteins may play a role in tumor invasion. CD44, a surface glycoprotein, can express Tn or sialilated (STn) antigens. It was shown with the use of genetically modified T-24 cells that Tn antigen overexpression on CD44 does not change invasion potential of T-24 cells in Matrigel invasion assay. On the other hand, STn-overexpression increased the number of T-24 cells invading Matrigel [43]. Increased migratory properties of T-24 cells vs. normal HCV-29 cells that were observed in the present study can also be attributed to protein sialilation. Although higher intensities of on-blot reactions with SNA and MAA lectins were observed for HCV-29 cells, surface expression of sialic acids observed in flow cytometry was higher for T-24 cells. Similar to fucosylation, sialilation of surface proteins, including CAMs, could have contributed to the higher migration rate of cancerous T-24 cells.

Far less is known about the role of EV glycosylation in cancer. As mentioned in Section 3.1, glycans are involved in protein sorting into EVs. There is also some evidence that EVs can impact EV biodistribution, uptake and effect exerted by EVs on recipient cells. For instance, desialilation of EVs with neuraminidase led to their increased accumulation in the lung and axillary lymph nodes of an EV-inoculated mouse in comparison to control EVs [21]. Similarly, EVs isolated from different murine hepatic cell lines after neuraminidase treatment displayed a slightly higher uptake by recipient cells, including ovarian cancer cells [19]. This was attributed to the decrease in negative charge at the EV surface after sialic acid removal and the exposure of other sugar residues at the EV surface that facilitated their binding and uptake.

EV glycosylation can also affect immune system response. It has been shown that macrophages expressing siglec-1 preferentially incorporate α-2,3-sialilated EVs [17]. This effect was observed neither in siglec-1-deficient mice nor for neuraminidase-treated EVs, suggesting that α-2,3-sialic acids in tumor-derived EVs can mediate a communication axis between immune and cancer cells. Moreover, the overexpression of high mannose type glycans in melanoma-derived EVs led to their increased uptake by dendritic cells and subsequent increase in CD8+ T cell response [44]. This was attributed to the binding of mannose residues to DC-SIGN, which is a C-type lectin receptor specific for dendritic cells. In addition, the enzymatic removal of sialic acids in glioblastoma-derived EVs led to their enhanced EV uptake by dendritic cells also in a DC-SIGN dependent manner, and the activation of both CD8+ and CD4+ T cell responses [45].

In the present study, the effect of protein glycosylation on EV biodistribution and uptake was not studied. However, we analyzed how altered glycosylation modulate the functional effect exerted by ectosomes on viability and migratory properties of recipient cells. Stronger pro-proliferative and promigratory activity of tumor-derived T-24 ectosomes was observed in comparison to ectosomes derived from normal HCV-29 cells. When added ectosomes were isolated from DMJ-treated cells, the aforementioned effect was diminished, suggesting that glycans carried by ectosomes are involved in modulation of recipient cell function. In analogous functional tests, HCV-29- and T-24-derived ectosomes were also shown to increase viability and motility of recipient endothelial HUVEC cells and Hs27 fibroblasts. This supports the hypothesis that ectosomes can modulate function of various cells present in the tumor microenvironment.

Based on lectin blotting and flow cytometry results, β1,6-branched complex type N-glycans (PHA-L staining) and N-glycans with α-2,6-linked sialic acids (SNA staining) were the most abundantly present in ectosomes and were most likely responsible for the observed functional effects. Further studies are needed to investigate the mechanism through which those glycans are involved in ectosome action. Ectosomal glycoproteins may be incorporated by recipient cells or directly transferred from ectosome to cell membrane without EV incorporation. Moreover, it is possible that only receptor–receptor interactions occur between ectosome surface glycoproteins and receptors on the recipient cell, followed by signal transduction.

Furthermore, glycosylation status of particular EV proteins may determine the effect that is exerted by EVs on recipient cancer cells. EVs from breast cancer cells induced invasiveness of recipient cells through the transfer of the extracellular matrix metalloproteinase inducer (EMMPRIN) [46]. The observed effect was dependent on the status of Asn 160 and Asn 268 EMMPRIN N-glycosylation sites. Similarly, EVs bearing CD82 protein inhibited the cell adhesion of recipient ovarian cancer cells, and no inhibition was observed when CD82 was not N-glycosylated at Asn 157 [47]. Lectin blotting results from the present study also suggest that the effect exerted by EVs on recipient cells may depend on the glycosylation status of particular proteins (or groups of proteins). For all six lectins, patterns of enrichment/depletion of bands were not uniform within the given ectosome samples, with some bands of higher intensities and some of lower intensities in comparison to the membrane fractions.

Finally, EVs can also transfer glycosyltransferases that modulate glycophenotype of the recipient cells. ST6Gal1 was identified in EVs that increased α-2,6-sialilation in the recipient cells [38]. Since α-2,6-sialilation is often correlated with increased tumor invasiveness, the transfer of ST6Gal1 and other glycosylation-related enzymes may be yet another mechanism by which EVs promote cancer progression.

### 3.3. Ectosomes as Carries of Cancer-Related and Clinically Relevant Glycoepitopes

There are several serological cancer markers relying on circulating glycans or glycoproteins, many of which display altered glycosylation. That includes the sialil Lewis A (CA19-9) and the STn (CA72-4) antigens, alpha-fetoprotein (AFP), prostate-specific antigen (PSA), mucin 16 (CA125), mucin 1 (CA15-3), carcinoembryonic antigen (CEA), and many more [48]. Circulating EVs are also heavily glycosylated and any alteration to the cell surface glycosylation may be reflected on the surface of EVs. That, together with the high accessibility and easy isolation from various body fluids, make EVs a potential target in the development of new diagnostic, prognostic, and therapeutic strategies in cancer.

For instance, higher levels of CA125 antigen were detected in blood EVs from ovarian cancer patients in comparison to detection in total serum [49]. Moreover, increased O-glycosylation was observed in EVs from pancreatic cancer patients, even from those negative for the CA19-9 antigen [50]. In another study, EVs from pancreatic cancer patients displayed elevated levels of CA19-9 in comparison to EVs from healthy controls [51]. In several cases, CA19-9 was detected in EVs even if the patient was previously determined as CA19-9-negative by total serum analysis. Heavily sialilated glycoisoform of prominin 1 (CD133) was also found in pancreatic cancer patients’ EVs and correlated with worse survival rate [52]. Glycosylated tenascin C in EVs from different tumor and non-tumor tissues was successfully used to distinguished them with high specificity and sensitivity [53]. Similarly, EMMPRIN was enriched in EVs from pancreatic tumor tissues but not in EVs from non-tumor tissues [53]. Further, glycosylated variants of EMMPRIN were detected in EVs from patients with different types of cancer and correlated positively with poor survival [54].

To our knowledge, there is only one study that concerned biomarker discovery in bladder cancer-derived EVs. With the use of a wide lectin panel, it has been found that fucosylated integrin α3 subunit (ITGA3) is enriched on the surface of EVs and can be detected by fucose-specific lectin UEA. It was later confirmed, using EVs from bladder cancer cells or cancer patient urine, that ITGA3-UEA biomarker combination can successfully discriminate bladder cancer patients from those with benign tumors and prostate cancer patients [55].

In the present study, glycosylation of particular ectosomal proteins was not analyzed. However, total glycosylation profile obtained by various methods can also be useful in search of novel biomarkers. For instance, reverse glycan capture strategy and liquid chromatography coupled with mass spectrometry was used to profile total N-glycome of hepatocellular carcinoma-derived exosomes [56]. The study revealed the enrichment in sialilated and fucosylated glycans in comparison to healthy controls. Moreover, glycan node analysis revealed significant differences between glycoprofiles of plasma-derived EVs and whole plasma [57]. Although only healthy donors were tested, the identification of specific differences in the glycosylation of plasma and plasma-derived EVs may serve as a reference for future diagnostic studies.

Furthermore, a new analytical platform i.e., integrated magnetic analysis of glycans in extracellular vesicles (iMAGE) was recently developed [58]. The platform utilizes magnetic nanoparticles to transduce EV-bound glycans into measurable magnetic signals after they are aggregated by lectin or antibody. In the study by Wang et al., [58] the use of Jacalin, ConA, RCA120, PHA-E, STA, LEL, WGA, DSL, and LCA lectins allowed to distinguish gastric and colorectal cancer patients according to their prognosis only by the glycans present on the EV surface.

In the present study, Western blot and flow cytometry were used to analyze glycoepitopes of HCV-29 and T-24 cells and ectosomes with a panel of six lectins (i.e., AAA, GNA, SNA, MAA, PHA-E, and PHA-L). Considering lectin blotting results, PHA-L and SNA stainings revealed the most differences between normal HCV-29 and T-24 cells, with higher expression of those epitopes in T-24 cells. Similarly, epitopes recognized by PHA-L and SNA lectins had higher expression in T-24-derived ectosomes than in HCV-29-derived ectosomes. Previous studies also pointed at the clinical relevance of β1,6-branching and α2,6-linked sialic acids in bladder cancer. However, the expression of N-acetylglucosamine transferase V (GnT-V) responsible for β1,6-branching expression in patient specimens was inversely correlated with tumor grade, disease stage, and disease-free survival [59]. This suggests differences between in vivo and vitro studies. Thus, EVs from patients samples should be analyzed in the future.

Regarding sialilation, elevated sialic acid levels are associated with worse prognosis in bladder cancer. Increased serum level of sialyl Lewis A has been correlated with higher stage, grade [60], and invasiveness of the tumor, whereas sialyl Lewis X with a shorter survival rate [61]. Furthermore, levels of total urinary sialic acids have been found to increase during the progression of bladder cancer [62]. Moreover, elevated total serum SA (SA) levels were proposed as potential biomarkers of localization of urothelial tumors (high grade tumor vs. upper urinary tract tumor) [63]. Finally, STn expression in both circulating tumor cells (CTC) and bladder cancer lesions promotes tumor dissemination and subsequent metastasis. A significant increase in STn antigen expression has been associated with high-grade lesions and decreased patient survival [64].

Lectin blotting, at β1,6-branched N-glycans and α2,6-linked sialic acids, for potential bladder cancer biomarkers is, however, a semiquantitative method with serious limitations regarding reproducibility. Moreover, a large amount of EVs in terms of protein concentration is required, making it difficult to obtain a clinical sample volume of blood or urine. On the other hand, the use of flow cytometry reduces the required sample volume, shortens the time of analysis, and allows for more high-throughput screening. In the present study, out of six stainings, only percentage of GNA-positive bladder cancer T-24 cells was higher than for HCV-29 cells. On the other hand, RFI measurements revealed significant differences for all six stainings. T-24 cells had a higher surface expression of all six analyzed glycoepitopes than HCV-29 cells. This points towards the potential use of this lectin panel in discrimination between normal and cancerous bladder epithelial cells. For ectosomes derived from HCV-29 or T-24 cells, no differences in the percentage of positive objects were observed. However, for PHA-E and MAA stainings, higher RFI was measured in the case of T-24-derived ectosomes. This suggests that bisecting GlcNAc and α2,3-linked sialic acids are the most promising glycobiomarker targets for ectosomes.

## 4. Materials and Methods

### 4.1. Reagents

Endothelial Cell Growth Medium (cat. C-22010), penicillin/streptomycin solution, trypsin-EDTA solution, Lumi-Light PLUS Western Blotting Kit (including anti-mouse and anti-rabbit IgG-HRP secondary antibodies), protease and phosphatase inhibitor cocktails, extravidin-alkaline phosphatase (AP) conjugate, and 1-deoxymannojirimycin (DMJ) were purchased from Merck Group (Darmstadt, Germany). RPMI 1640 GlutaMAX™-I medium, DMEM medium, foetal bovine serum (FBS), Alamar Blue cell viability reagent, and MicroBCA Protein Assay kit were obtained from Thermo Fisher Scientific (Waltham, MA, USA). The 2x concentrated Laemmli Buffer was obtained from Bio-Rad Laboratories (Hercules, CA, USA). Biotinylated lectins: *Phaseolus vulgaris* leukoagglutinin (PHA-L), *Phaseolus vulgaris* erythroagglutinin (PHA-E), *Sambucus nigra* agglutinin (SNA), *Maackia amurensis* agglutinin (MAA), *Galanthus nivalis* agglutinin (GNA), and *Aleuria aurantia* agglutinin (AAA) were purchased from Vector Laboratories Inc. (Burlingame, California, United States). ApogeeMix reference beads were purchased from Apogee Flow Systems (Northwood, UK). Polyvinyidene fluoride membranes (PVDF) were from Millipore (Burlington, MA, USA). All remaining chemicals were of analytical grade, commercially available.

### 4.2. Antibodies

The following monoclonal primary antibodies were used for characterization of ectosomes samples: anti-CD63 mouse monoclonal primary antibody (clone RFAC4, cat. CBL553) from Sigma-Aldrich (St. Louis, MO, USA), mouse monoclonal primary antibodies for Arf6 (clone 3A-1, cat. sc-7971), and Hsp70 (clone C92F3A-5, cat. sc-66048) from Santa Cruz Biotechnology (Dallas, TX, USA).

The following primary antibodies were used for characterization of membrane fraction purity: anti-GAPDH mouse polyclonal antibody and anti β-actin mouse monoclonal antibody (clone AT-15) from Sigma-Aldrich (St. Louis, MI, USA), anti-α5 integrin (clone SAM-1), and anti-β1 integrin (clone B3B11) mouse monoclonal antibodies from Merck Millipore (Darmstadt, Germany) and anti-histone 2B rabbit monoclonal antibody (clone IGX4228R-1) from abcam (Cambridge, UK).

### 4.3. Cell Lines

Human bladder cancer cell line (T-24) [65] and human non-malignant ureter epithelium cell line (HCV-29) [66] were kindly donated by Prof. Danuta Duś of the Institute of Immunology and Experimental Therapy of the Polish Academy of Sciences, Wroclaw, Poland. Human foreskin fibroblast cell line (Hs27) was kindly provided by Assoc. Prof. Halina Jurkowska of the Faculty of Medicine of the Jagiellonian University Medical College, Krakow, Poland. Human umbilical vein endothelial cells (HUVECs, cat. C-12203) were purchased from Merck Group (Darmstadt, Germany).

### 4.4. Culture Conditions and Cell Treatment

T-24 and HCV29 cells were cultured in RPMI 1640 medium with GlutaMAX-I, supplemented with 10% FBS and antibiotics: penicillin (100 unit/mL) and streptomycin (100 μg/mL). Hs27 cells were grown in DMEM medium supplemented with 10% FBS and antibiotics: penicillin (100 unit/mL) and streptomycin (100 μg/mL). HUVECs were maintained in dedicated Endothelial Cell Growth Medium. All cells were kept at 37 °C with 5% CO_2_ in a humidified incubator and passaged after reaching ~80% confluence.

Experiments were initiated after T-24 and HCV-29 cells reached sub-confluence. Then, the cells were cultured for 24 h in serum-free media prior to collecting cells and conditioned media for further experiments. In some experiments, these cells were grown for 24 h in serum-free medium containing additionally 1 mM DMJ.

### 4.5. Preparation of the Whole Cell Protein Extracts

The whole cell protein extracts were prepared as described previously [36]. In brief, T-24 and HCV-29 cells after 24 h of growth in serum-free media were harvested with a rubber policeman and pelleted. Then, the cells were sonicated on ice in 50 mM Tris/HCl buffer, pH 7.5, containing 150 mM NaCl, 2 mM EDTA, protease inhibitor cocktail (2 μL/100 µL), phosphatase inhibitor cocktail (1 µL/100 µL), 1 mM phenylmethylsulfonyl fluoride, 1 mM sodium fluoride and 5 mM tetrasodium pyrophosphate, and incubated with the same buffer containing additionally 0.3% protamine sulfate and 1% Triton X-100 (for 1 h, on ice). The whole cell protein extracts were then clarified by 18,000× *g* centrifugation for 20 min at 4 °C.

### 4.6. Isolation of the Membrane Protein Fraction and Assessment of Its Purity

The membrane protein fractions of T-24 and HCV-29 cells were obtained using Qproteome Cell Compartment Kit and desalted via acetone precipitation according to the manufacturer’s protocol.

The membrane fraction purity was evaluated by Western blot as described previously [36]. In brief, equal amounts of protein (70 µg according to MicroBCA method) from the whole cell extracts and the membrane protein fractions were mixed 1:1 with 2x concentrated Laemmli Buffer and separated by 10% sodium dodecyl sulfate-polyacrylamide gel electrophoresis (SDS-PAGE) under reducing conditions and then transferred to the PVDF membrane in buffer containing 0.25 mM Tris, 0192 M glycine, and 20% methanol, pH 8.4. Next, blots were blocked in 3% BSA in TBS/Tween (20 mM Tris/HCl, pH 7.6, 150 mM NaCl and 0.1% Tween 20). Then, the membranes were incubated with primary antibodies to α5 and β1 integrin subunits (dilutions 1:5000, markers for the membrane fraction), β-actin (dilution 1:10,000, marker for the cytoskeletal fraction), GAPDH (dilution 1:4000, marker for the cytosolic fraction), and histone 2B (dilution 1:500, marker for the nuclear fraction) and then with suitable anti-mouse IgG-HRP or anti-rabbit IgG-HRP (dilution 1:4000). Visualization of proteins was achieved with the use of HRP substrates from Lumi-Light PLUS Western Blotting Kit and ChemiDoc Imaging System (Bio-Rad).

### 4.7. Ectosome Isolation and Assessment of the Ectosome Samples Purity

Collected conditioned media were subjected to sequential centrifugation as described previously [32]. In brief, after three initial centrifugation steps, final centrifugation at 18,000× *g* for 20 min at 4 °C allowed to pellet ectosomes that were resuspended in ice-cold PBS.

Then, the purity of ectosome samples was evaluated by transmission electron microscopy (TEM) as well as by nanoparticle tracking analysis (NTA) as described previously in [36] and [32], respectively. Western blot analysis of EV markers was performed as previously described [32]. In brief, the whole cell protein extracts and ectosome samples containing 50 μg of protein (according to MicroBCA method) were mixed 1:1 with 2x concentrated Laemmli Buffer and separated by 10% SDS-PAGE under reducing conditions and then transferred to the PVDF membrane. After being blocked with 3% BSA in TBS/Tween, blots were probed with primary antibodies to CD63 (dilution 1:2000), Hsp70 (dilution 1:2000), and Arf6 (dilution 1:500) and then with anti-mouse IgG secondary antibody (dilution 1:400) conjugated to horseradish peroxidase (HRP). The signals were detected using HRP substrates from Lumi-Light PLUS Western Blotting Kit and ChemiDoc Imaging System (Bio-Rad).

### 4.8. Comparative Analysis of Cell and Ectosome Glycosylation

#### 4.8.1. Lectin Blotting

Lectin blotting was performed as described in [67]. In brief, material obtained from cultures of T-24 and HCV-29 cells previously maintained for 24 h in serum-free medium (i.e., the whole cell extracts, membrane protein fractions, and ectosome samples) containing an equal amount of protein (70 µg according to MicroBCA method) was separated by 10% SDS-PAGE under reducing conditions and transferred to the PVDF membrane. Afterwards, the membranes were incubated in Carbo Free blocking solution, probed with biotinylated PHA-E, PHA-L, MAA, SNA, AAA, and GNA lectins, and finally with extravidin-AP conjugate. The conjugated AP was detected via NBT/BCIP staining. Simultaneously, the samples were incubated with lectins preincubated overnight with the competitive sugar or CH_3_COOH (details on blocking solution in Supplementary Data, Table S1).

#### 4.8.2. Flow Cytometry

Surface glycosylation of untreated or treated with DMJ T-24 and HCV-29 cells as well as of ectosomes derived from those cells was analyzed by flow cytometry with the use of lectins as described in [36]. In brief, cells (7 × 10^4^) and ectosomes (isolated form 10 mL of conditioned media per sample) were incubated for 45 min on ice with biotinylated PHA-E, PHA-L, MAA, SNA, AAA and GNA lectins (20 μg/mL PBS). Afterwards, cells and ectosomes were washed in PBS and incubated with fluorescein isothiocyanate (FITC)-extravidin conjugate (25 μL/mL) under the same conditions and washed again. Cells were assessed for fluorescence in a FACS Calibur flow cytometer (BD Biosciences, San Diego, CA, USA). For each sample a total of 10^4^ cells was analyzed. Ectosome samples were analyzed on Apogee A50 Micro flow cytometer (Apogee Flow Systems, Hemel Hempstead, UK) for 120 s with flow rate of 1.5 μL/min, sample volume set at 150 μL and sheath pressure at 150 mb. The trigger was set on MALS detector with 420 V voltage, and on green fluorescent channel (488Gn) with 475 V voltage on 488 nm laser. For calibration, the reference beads composed of a mixture of 180 nm, 240 nm, 300 nm, 590 nm, 880 nm, and 1300 nm silica vesicles and 110-nm and 500-nm green fluorescent latex beads were used. For the analysis, samples were diluted in order to obtain < 5000 events/s.

### 4.9. Functional Tests

#### 4.9.1. Alamar Blue Cell Viability Assay

The Alamar Blue assay was performed according to the manufacturer’s protocol. In brief, T-24, HCV-29, HUVEC and Hs27 cells were seeded onto 96-well plates at the density of 1 × 10^4^ cells/100 µL. The next day, the culture medium was changed to serum-free medium, and cells were incubated with T-24- or HCV29-derived ectosomes (60 µg of protein) for 18 h. Then, 10% of Alamar Blue reagent was added to each well for 2 h and fluorescence intensity was measured at 560/595 nm. Results were standardized in relation to the untreated control (cells without addition of ectosomes), taken as 1.

#### 4.9.2. Wound Healing Assay

Wound healing assay was performed as described previously [68]. In brief, T-24, HCV-29, HUVEC, and Hs27 cells were grown to confluence on 6-well plates. Then, the medium was removed, and cell-coated surfaces were scraped with a 200 μL pipette tip. Next, T-24- and HCV29-derived ectosomes (60 µg of protein) were added to each well and the wounded areas were allowed to heal for 18 h at 37 °C. Based on multiple measurements of the width of the wounds at 0 h and 18 h, the degree of wound closure was estimated. Results were standardized in relation to the untreated control (cells without addition of ectosomes) taken as 1.

### 4.10. Other Methods

The protein concentration was determined with the use of a MicroBCA Protein Assay Kit according to the manufacturer’s protocol using bovine serum albumin (BSA) as standard. According to Laemmli, 10% SDS-PAGE was performed [69].

### 4.11. Statistical Analysis

Alamar Blue and wound healing assays as well as lectin blotting and flow cytometry were performed in triplicate for each experimental conditions. The results were presented as mean ± standard deviation. Analysis of variance (one-way ANOVA) and post-hoc Tukey’s test were performed with the use of Statistica 12 software. A *p*-value of <0.05 was considered statistically significant.

## 5. Conclusions

Bladder cancer is a malignancy that remains a therapeutic challenge and requires the identification of new biomarkers and mechanisms of progression. In the present study, we compared glycosylation profiles of T-24 urothelial bladder cancer cells, HCV-29 normal ureter epithelial cells, and ectosomes released by both cell lines. We showed that ectosomes from normal and cancerous cells possess distinct total and surface glycosylation profiles with an abundance of β-1,6-branched glycans and sialilated structures. Glycoepitopes identified in tumor-derived ectosomes might also be responsible for their cancer-promoting effect exerted on normal epithelial cells, endothelial cells, and fibroblasts. Further investigation on glycoproteins present in bladder cancer-derived EVs should focus on their precise role in cancer-associated biological processes and their applicability as novel biomarkers of the disease.

## Data Availability

Not applicable.

## Supplementary Materials

The following supporting information can be downloaded at: https://www.mdpi.com/article/10.3390/ijms232214368/s1.

## Abbreviations

AAA	*Aleuria aurantia* agglutinin
BCIP	5-bromo-4-chloro-3-indolyl-phosphate
CAMs	cell adhesion molecules
EVs	extracellular vesicles
GAPDH	glyceraldehyde 3-phosphate dehydrogenase
GNA	*Galanthus nivalis* agglutinin
MAA	*Maackia amurensis* agglutinin
NTA	Nano Tracking Analysis
NBT	4-nitro blue tetrazolium chloride
PHA-E	*Phaseolus vulgaris* erythroagglutinin
PHA-L	*Phaseolus vulgaris* leukoagglutinin
SDS-PAGE	sodium dodecyl sulfate polyacrylamide gel electrophoresis
SNA	*Sambucus nigra* agglutinin
TACA	tumor-associated carbohydrate antigen
TEM	transmission electron microscopy
