# Supplementary material for: Lectin-Based Study Reveals the Presence of Disease-Relevant Glycoepitopes in Bladder Cancer Cells and Ectosomes
Source: Int J Mol Sci. 2022 Nov 19;23(22):14368. doi: 10.3390/ijms232214368 (PMC9699364; doi:10.3390/ijms232214368)
Supplement: Supplementary file 1 [file ijms-23-14368-s001.zip › ijms-2028803-supplementary.pdf]

Supplementary Data: Lectin blotting results

Methodology: Lectin blotting was performed as described in [67]. In brief, material obtained from cultures of T-24 and HCV-29 cells previously maintained for 24 h in serum-free medium (i.e. the whole cell extracts, membrane protein fractions and ectosome samples) containing equal amount of protein (70 μg according to MicroBCA method) was separated by 10% SDS-PAGE under reducing conditions and transferred to the PVDF membrane. Afterwards, the membranes were incubated in Carbo Free blocking solution, probed with biotinylated PHA-E, PHA-L, MAA, SNA, AAA and GNA lectins and finally with extravidin-AP conjugate. The conjugated AP was detected via NBT/BCIP staining Simultaneously, the samples were incubated with lectins preincubated overnight with the competitive sugar or CH_3COOH.

Table S1. Sugar-binding specificities of lectin used for lectin blotting studies and composition of blocking solutions.

Lectin	Lectin-specific glycan structures	Blocking solution
Aleuria aurantia agglutinin (AAA)	Fucα1,6GlcNAc-Asn<br>Fucα1,2Galβ1,4GlcNAcGalβ1,4(Fucα1,3)GlaNAc-	0.5 M L-(-)-fucose)
Galanthus nivalis agglutinin (GNA)	Manα1,2Man-<br>Manα1,6Man-<br>Manα1,3Man-	0.5 M methyl-α-Dmannopiranoside
Sambucus nigra agglutinin (SNA)	NeuAcα2,6Gal-	0.4 M CH_3COOH
Maackia amurensis agglutinin (MAA)	NeuAcα2,3Gal-	0.4 M CH_3COOH
Phaseolus vulgaris leukoagglutinin (PHA-L)	Galβ1,4GlcNAcβ1,6Manα-<br>Galβ1,4GlcNAcβ1,2Manα-<br>(β1,6-branched structures of N-glycans)	1 M N-acetyl-Dgalactosamine
Phaseolus vulgaris erythroagglutinin (PHA-E)	Galβ1,4GlcNAcβ1,2Man (the bisecting GlcNAcβ1,4Man is essential)	1 M N-acetyl-Dgalactosamine

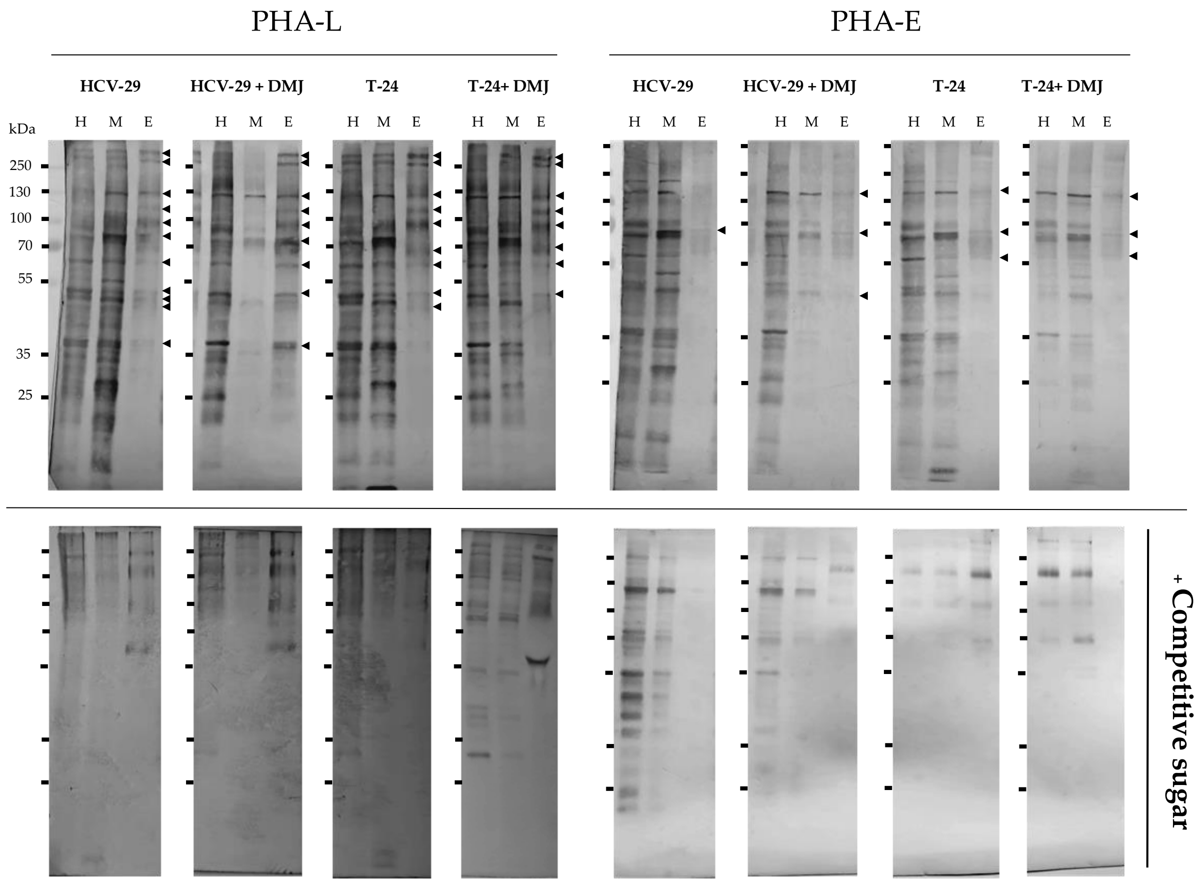

Figure S1. Detection of glycoproteins possessing β1,6-branched tri- and/or tetraantennary complex type N-glycans (PHA-L-positive) and bisecting GlcNAc bound to the core mannose of complex type N-glycans (PHA-E-positive). Results of lectin blotting with PHA-L and PHA-E are shown in the upper panels. Controls with the competitive sugars are shown in the lower panels. The major specific bands were indicated on the right with arrowheads. H, the whole cell protein extract; M, the membrane fraction; E, the ectosomal fraction.

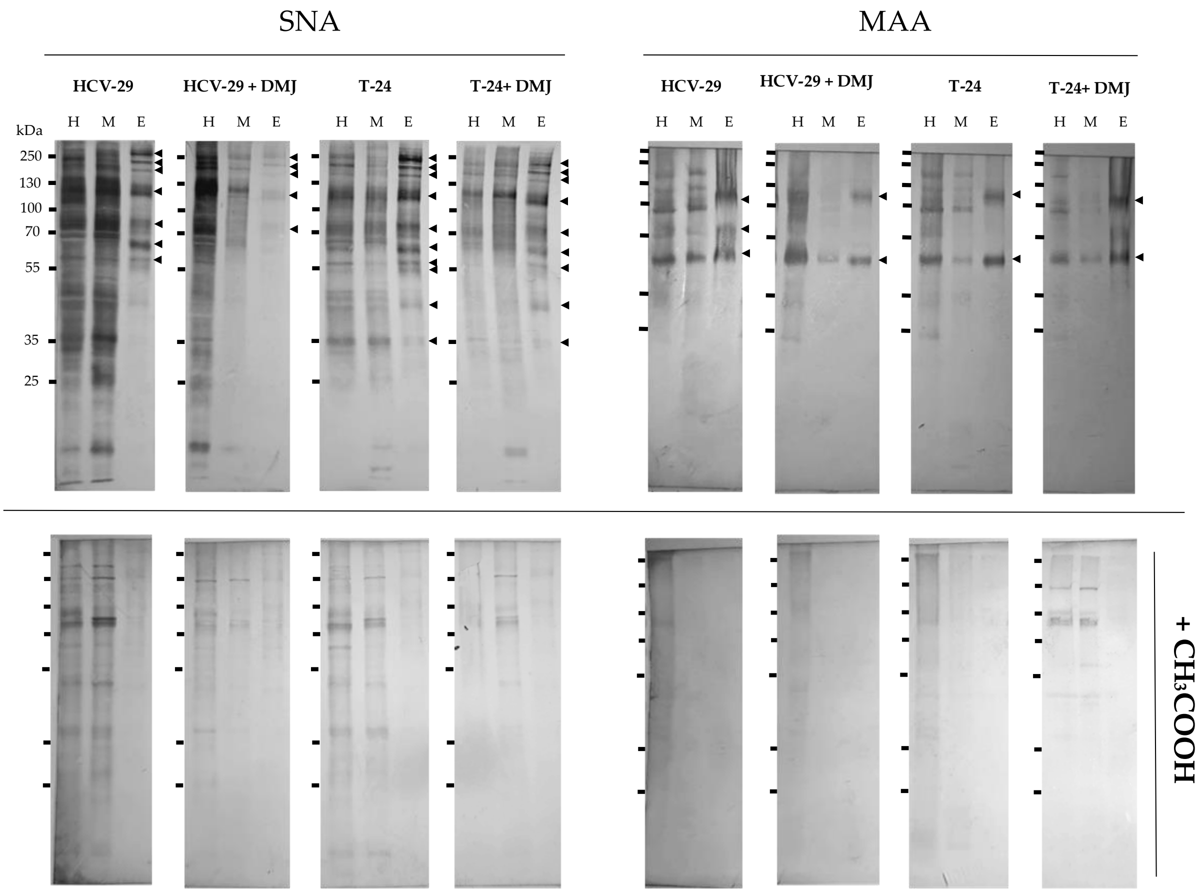

Figure S2. Detection of glycoproteins carrying α2,6-linked (SNA-positive) and α2,3-linked (MAA-positive) sialic acids. Results of lectin blotting with SNA and MAA are shown in the upper panels. Controls with the competitive sugars are shown in the lower panels. The major specific bands were indicated on the right with arrowheads. H, the whole cell protein extract; M, the membrane fraction; E, the ectosomal fraction.

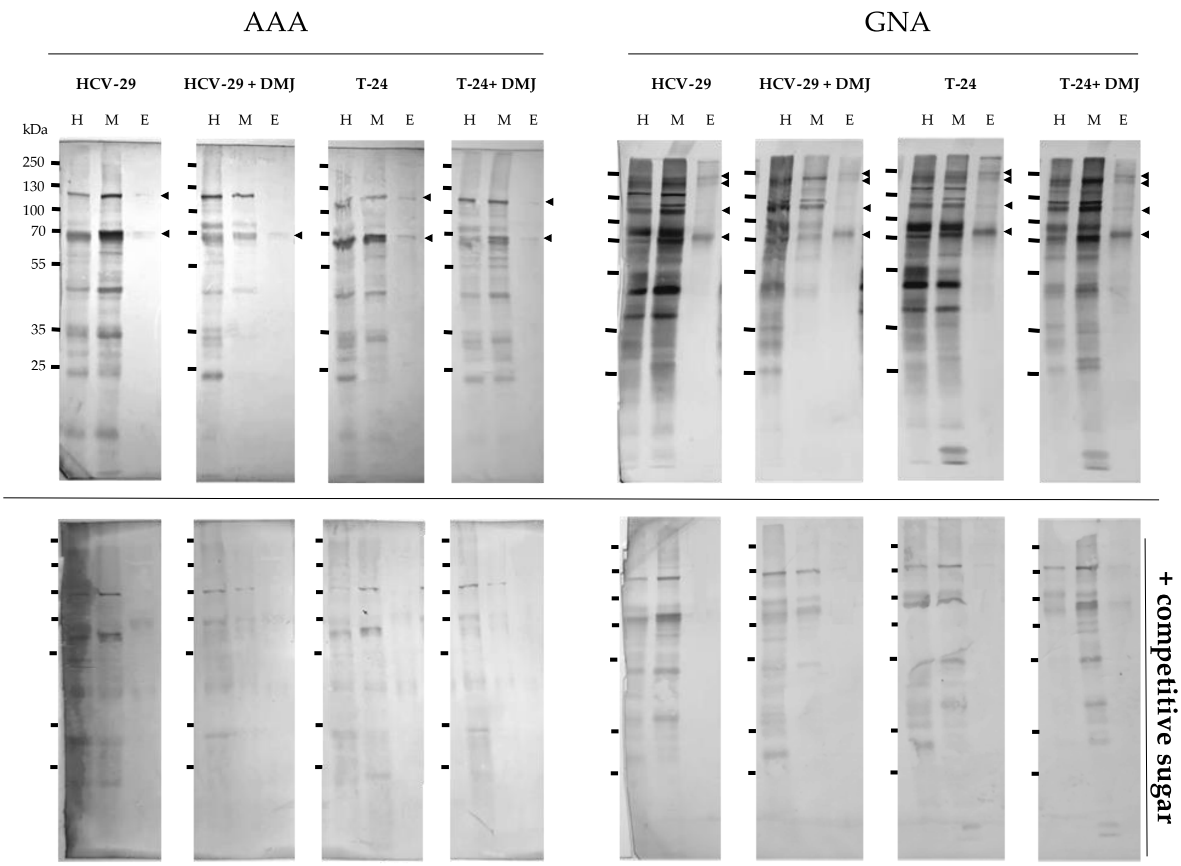

Figure S3. Detection of glycoproteins having fucose (AAA-positive) and mannose (GNA-positive) residues. Results of lectin blotting with AAA and GNA are shown in the upper panels. Controls with the competitive sugars are shown in the lower panels. The major specific bands were indicated on the right with arrowheads. H, the whole cell protein extract; M, the membrane fraction; E, the ectosomal fraction.
